# Detecting local heterogeneity and ionization ability in the head group region of different lipidic phases using modified fluorescent probes

**DOI:** 10.1038/srep08699

**Published:** 2015-03-03

**Authors:** Osama K. Abou-Zied, N. Idayu Zahid, M. Faisal Khyasudeen, David S. Giera, Julian C. Thimm, Rauzah Hashim

**Affiliations:** 1 Department of Chemistry, Faculty of Science, Sultan Qaboos University, P.O. Box 36, Postal Code 123, Muscat, Sultanate of Oman; 2 Department of Chemistry, Faculty of Science, University of Malaya, 50603 Kuala Lumpur, Malaysia; 3 Glycoteam GmbH, Martin-Luther-King-Platz 6, D-20146 Hamburg, Germany

## Abstract

Local heterogeneity in lipid self-assembly is important for executing the cellular membrane functions. In this work, we chemically modified 2-(2′-hydroxyphenyl)benzoxazole (HBO) and attached a C_8_ alkyl chain in two different locations to probe the microscopic environment of four lipidic phases of dodecyl *β*-maltoside. The fluorescence change in HBO and the new probes (HBO-1 and HBO-2) shows that in all phases (micellar, hexagonal, cubic and lamellar) three HBO tautomeric species (solvated *syn*-enol, anionic, and closed *syn*-keto) are stable. The formation of multi tautomers reflects the heterogeneity of the lipidic phases. The results indicate that HBO and HBO-1 reside in a similar location within the head group region, whereas HBO-2 is slightly pushed away from the sugar-dominated area. The stability of the solvated *syn*-enol tautomer is due to the formation of a hydrogen bond between the OH group of the HBO moiety and an adjacent oxygen atom of a sugar unit. The detected HBO anions was proposed to be a consequence of this solvation effect where a hydrogen ion abstraction by the sugar units is enhanced. Our results point to a degree of local heterogeneity and ionization ability in the head group region as a consequence of the sugar amphoterism.

Cellular functions such as signaling, adhesion, motility and membrane trafficking may be understood through the concept of lipid raft and lipid domain heterogeneity in cell membrane^1^,^2^,^3^,^4^,^5^. Instead of a simple homogeneous fluid bilayer assumed previously^6^, a cell membrane has different structural partitions with different degrees of lipid lateral organizations (e.g. liquid-ordered, liquid-disordered and gel)^7^,^8^,^9^. In a model membrane experiment, it was reported that lysenin recognizes the heterogeneous organization of biomembranes which was modified by the presence of glycolipids^10^.This suggest heterogeneity is more pervasive^11^, and may be inherited from the chemical properties of the constituent lipids, which have the ability to bind to different molecular structures. For example, the pH in the vicinity of the membrane environment may reflect the local heterogeneity which influences the ionization state of neighbouring molecules such as drugs^12^,^13^,^14^. Unionized drugs are lipid soluble and diffusible, able to cross the membrane's lipid bilayer, unlike the charged species^15^,^16^,^17^. However, most drugs can be classified in general as either weak acids or weak bases^18^. Since a lipid may ionize or protonate small molecules, this will influence the drug's neutral state and its ability to cross the membrane. Therefore, it is important to detect the degree of ionization of a drug molecule as a consequence of the local effect of the surrounding lipids (pH and polarity) and this justifies a systematic study in order to better understand the lipid-drug interaction^18^,^19^,^20^,^21^.

We have recently examined in detail some glycolipid self-assembly systems using several fluorescent probes^22^,^23^,^24^. We reported a polarity gradient in the head group region of different glycolipid phases. Using tryptophan and its alkyl esters as probes, we found the polarity to be similar to that of simple alcohols when the tryptophan moiety is close to the aqueous medium. The polarity was similar to that of dioxane when the probe is located deep inside the head group domain^22^. In the present work, we investigate the ability of the lipid self-assembly to stabilize different chemical structures of a ligand and the ability to affect the ligand's neutral state. In order to achieve this goal, we employed 2-(2′-hydroxyphenyl)benzoxazole (HBO, shown in Figure 1(a)) as a fluorescent probe. By keeping the HBO moiety unchanged, we placed its OH group in different locations within the lipid head group region by attaching a C_8_ alkyl chain to the benzoxazole ring (HBO-1) or to the phenyl ring (HBO-2). A sketch of the new compounds is shown in Figure 1(b).

HBO belongs to the benzazoles family^25^,^26^. In several enzymatic reactions different benzazole derivatives were reported to have different inhibitory activity and are proposed to treat some diseases^27^,^28^. This unique inhibitory activity is due to the N-heteroatoms in the benzazoles acting as both proton donor and/or acceptor^29^. Figure 1(a) shows the different tautomeric forms of the HBO molecule^30^,^31^,^32^,^33^,^34^,^35^,^36^,^37^. In the ground state, the molecule exists in a conformational equilibrium between the *syn*- and *anti*-enols. In addition, the phenoxy group of *syn-*enol may form an internal H-bond (closed *syn*-enol) or an intermolecular H-bond with a solvent molecule (solvated *syn*-enol). Only the closed *syn*-enol efficiently forms the closed *syn*-keto tautomer upon photoinduced excitation (excited-state intramolecular proton transfer (ESIPT)). In non-H-bonding solvents, such as cyclohexane, which are unable to effectively compete for the phenolic proton, excitation results in very efficient ESIPT and long-wavelength fluorescence. However, in protic solvents, which are more able to compete for the phenolic proton, emission from both enol and keto are observed with intensities that depend on the concentration of the closed *syn*-enol relative to the solvated *syn* and *anti*-enols.

In aqueous medium, on the other hand, we have shown that the formation of the open *syn*-enol tautomer that is solvated by a network of two water molecules is more favorable (see Figure 1(a))^37^. Upon excitation, the open *syn*-enol undergoes an efficient intermolecular proton transfer in the excited state to yield the open *syn*-keto tautomer. In addition to the neutral forms of HBO, we detected the anion form in basic medium and in binary solvents^37^.

The systems under study are lipidic phases from the industrially produced dodecyl *β*-maltoside (*β*Mal-C_12_), a glycolipid which has been used in the purification and stabilization of proteins, like RNA polymerase, and the detection of protein-lipid interactions^38^,^39^. The maltoside sugar is sufficiently large for the fluorescence molecule to be probed in the head group region^22^. The micellar, hexagonal, cubic (*Ia*3*d* space group) and lamellar phases are all in normal (type I) state and were obtained at a specific concentration and temperature of aqueous formulation of *β*Mal-C_12_, as reported in its phase diagram^40^.

## Results and Discussion

### Spectroscopic Characterization of the HBO Probes in Solution

The absorption spectra of HBO in different solvents and in aqueous pH 12.0 are shown in Figure 2(a). In neutral solvents, the absorption peaks in the region 315–340 nm are attributed to the π* ← π transitions of the *anti*-enol (the blue peak) and the *syn*-enol (the red peak)^30^. A hypsochromic shift and less structured peaks are clearly shown for protic solvents due to intermolecular hydrogen-bonding interactions between the solvent and the hydrogen bonding sites of HBO^31^,^32^,^37^. In water, each hydrogen bonding site in HBO (*anti* and *syn* tautomers) is solvated by two water molecules that are in direct contact with the OH group and the N/O heteroatom^37^. This particular solvation mechanism is supported by the stable planarity of the HBO backbone^41^,^42^.

In aqueous pH 12.0, the absorption band is red-shifted by ~1450 cm^−1^ and is very broad with no structural features. This observation points to a larger degree of π conjugation upon deprotonation of HBO. This is expected since losing the hydroxyl proton diminishes the intramolecular hydrogen bond and allows the negative charge over the oxygen atom to participate in the resonance structure of the phenyl ring, giving it more aromaticity and a more single character to the bond connecting the two rings.

**Figure 1:**
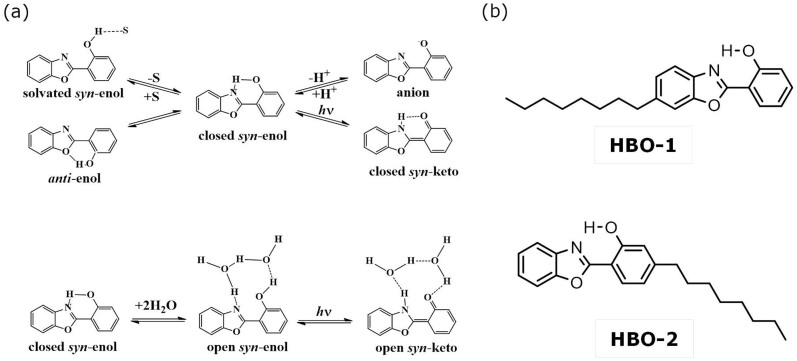
(a) Tautomeric forms of HBO. S represents polar solvents. (b) Chemical structures of the new probes.

**Figure 2:**
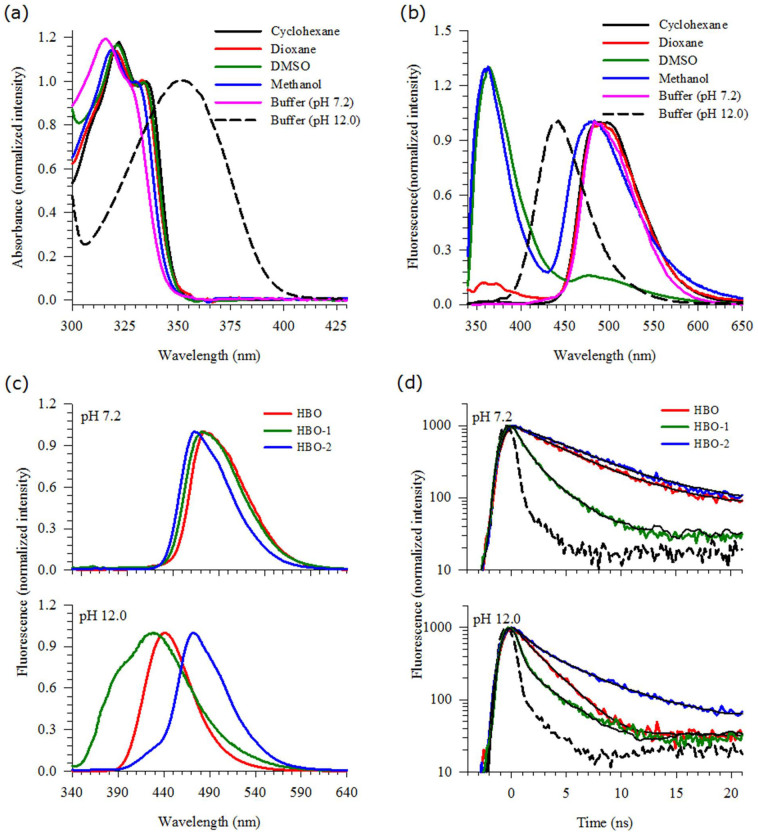
(a) Absorption spectra of HBO in different solvents, showing the lowest-energy band. (b) Fluorescence spectra of HBO in different solvents. λ_ex_ = 330 nm. (c) Fluorescence spectra of HBO and its derivatives in different pH solutions. λ_ex_ = 330 nm. (d) Fluorescence decay transients of HBO and its derivatives in aqueous solutions of different pH values. λ_ex_ = 340 nm. Signal was measured using a 380 nm cut-off filter. IRF is shown in a dashed line. Black solid lines represent the best fits.

The corresponding fluorescence spectra are shown in Figure 2(b). For HBO in cyclohexane, the broad Stokes-shifted peak in the region 480–500 nm is due to the *syn*-keto tautomer^31^,^37^. This result reflects the efficient ESIPT process in a nonpolar solvent that does not interfere with the intramolecular hydrogen bond. A similar fluorescence result was obtained in dioxane with a small contribution of a second peak centered at ~370 nm. The latter is due to fluorescence from the *anti*- and/or solvated *syn*-enols (see Figure 1(a))^31^. In DMSO, on the other hand, fluorescence due to the *syn*-keto tautomer of HBO is depressed and most of the fluorescence intensity is located at 370 nm. As a highly polar, aprotic solvent, DMSO is expected to strongly interact with the OH group of the *syn*-enol tautomer, thus stabilizing the solvated structure of this tautomer.

In methanol, fluorescence of HBO shows two peaks that are centered at 370 and 480 nm. The latter is due to the closed *syn*-keto tautomer which is supported by the fluorescence lifetime measurements^30^,^31^,^32^. In aqueous medium, on the other hand, the tendency of water molecules to strongly associate with each other through intermolecular hydrogen bonds allows more than one molecule of water to form a solvent network that facilitates intermolecular proton transfer with the solute^43^. In a previous study, we found that two water molecules solvate the hydrogen-bonding site of the *syn*-enol tautomer in which HBO undergoes water-assisted tautomerization in the excited state to yield the open *syn*-keto (see Figure 1(a))^37^. The peak at ~490–500 nm is a manifestation of this mechanism with a fluorescence lifetime that is one to two orders of magnitude longer than that in methanol^44^,^45^. In basic aqueous medium, the only peak in the spectrum is at 445 nm which is assigned to the anion form of HBO^37^,^44^,^45^.

Since all the lipidic systems studied here are composed of a high percentage of water, it is important to understand the fluorescence behavior of HBO, HBO-1 and HBO-2 in aqueous medium before the study is carried out in lipids. From the above results and from our previous study on HBO^37^,^44^,^45^, fluorescence in neutral and basic aqueous media is simple with only one species dominating in each case as shown above. We carried out a fluorescence study on aqueous solutions of HBO-1 and HBO-2 in order to investigate the effect of the long alkyl chain attached on each side of the parent HBO molecule. Figure 2(c) displays the results in pH 7.2 and 12.0. In neutral pH, the fluorescence behavior in general is similar to that of HBO, except for a slight blue shift of the keto fluorescence peak which is more pronounced in HBO-2. On the other hand, in basic aqueous medium the fluorescence is different for each derivative. Unlike HBO where there is a complete anion formation in pH 12.0 (peak at ~440 nm), the fluorescence behavior of HBO-1 shows an additional blue peak in the 380 nm region which coincides with either the *anti*-enol or the solvated *syn*-enol. In HBO-2, the keto tautomer dominates the fluorescence signal in pH 12.0 (peak at ~470 nm) with a small contribution from the anion species.

Fluorescence decay transients for HBO and its derivatives are shown in Figure 2(d) for aqueous solutions of pH 7.2 and 12.0. The lifetime data are summarized in Table 1. In neutral pH, both HBO and HBO-2 show lifetime values (τ_1_) that are consistent with the formation of the open *syn*-keto (water-assisted tautomerization)^44^,^45^. The results show that the fluorescence lifetime of the open *syn*-keto is much longer than the lifetimes in other solvents where the dominant form is the closed *syn*-keto^31^,^32^,^34^,^35^,^36^,^43^,^44^,^45^. This is attributed to the local solvation of the hydrogen bonding center by a closed-water network^37^. A similar trend has been reported for other systems in which the involvement of water molecules in the tautomerization dynamics stabilizes the molecule in the excited state^36^,^46^,^47^. In contrast, the lifetime results for HBO-1 in neutral pH show two components of 0.8 and 2.4 ns. According to the previous assignments of the fluorescence lifetimes for HBO in different solvents^31^,^32^,^34^,^35^,^36^,^44^,^45^, the short component is due to contribution from a closed *syn*-keto tautomer (τ_2_) and the long component is the fluorescence decay of the anion species (τ_3_). It is important to mention here that HBO-1 does not favor the formation of an open *syn*-keto, in contrast to both HBO and HBO-2. A strong intramolecular hydrogen bond may be the reason that solvation of HBO-1 by water is not strong enough to break the intramolecular hydrogen bond in the molecule.

In aqueous solution of pH 12.0, HBO shows only one lifetime component in the fluorescence decay transient which was assigned to the anionic form (2.6 ns)^37^. For HBO-2, the dominant open *syn*-keto peak in the steady-state fluorescence spectrum dominates the decay transient (6.1 ns, 74%) in addition to a small contribution from the anion species (1.3 ns, 26%). Both the steady-state and time-resolved fluorescence results indicate that, unlike HBO, HBO-2 is not easy to ionize. On the other hand, the transient decay for HBO-1 in pH 12.0 shows two components of 0.4 and 2.8 ns. The latter component is a typical lifetime of the anion species, while the former coincides with the formation of a solvated *syn*-enol^31^,^32^. Accordingly, the fluorescence shoulder in the 380 nm region is due solely to the neutral *syn*-enol form of HBO-1 with no contribution from the *anti*-enol tautomer. In a previous study, we have shown that the lifetime of the *anti*-enol tautomer is ~1.5 ns and solvent-independent^31^.

The above results serve as a benchmark for understanding the local environment in lipids when HBO and its derivatives are used as probes. We will next examine the change in the fluorescence spectra and decay transients of HBO, HBO-1 and HBO-2 when they are incorporated in different lipidic phases.

### Stability of Specific Tautomers of HBO in Lipids

**Figure 3:**
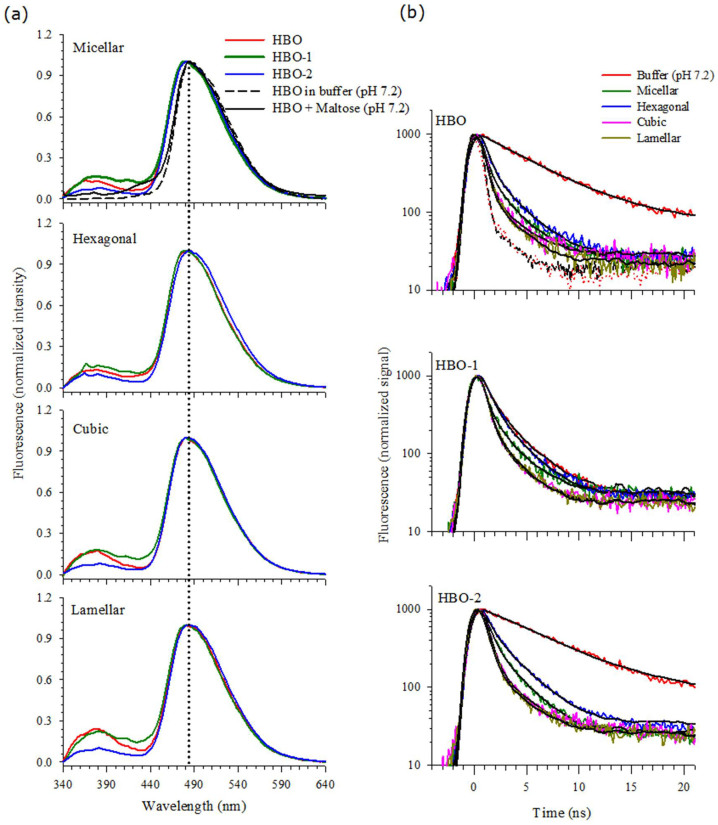
Fluorescence spectra of HBO and its derivatives. (a) in different phases of the *β*Mal-C_12_ lipid. The corresponding spectra of HBO alone and mixed with maltose (1:1 molar ratio) in buffer are shown in the upper segment for comparison. λ_ex_ = 330 nm. (b) Fluorescence decay transients of HBO and its derivatives in lipids. The decay transients of HBO and its derivatives in buffer are included for comparison. λ_ex_ = 340 nm. Signal was measured using a 380 nm cut-off filter. IRF is shown in a dashed line. Black solid lines represent the best fits. Signal from lipid only is shown by red dots which matches the IRF signal.

The fluorescence spectra of HBO, HBO-1 and HBO-2 incorporated inside four lyotropic self-assemblies namely micellar, hexagonal, cubic and lamellar phases are shown in Figure 3(a) for λ_ex_ = 330 nm. The spectrum of HBO in buffer is included for comparison in the upper segment of the graph. In all the lipidic phases, a major peak for the *syn*-keto tautomer around 480 nm is evident. Unlike HBO in buffer, a small contribution from the solvated *syn*-enol and/or the *anti*-enol tautomers is observed in all lipids in the region 370–380 nm. Contribution from the latter is more for HBO-1, followed by HBO then HBO-2. In addition to these tautomers, there is a small contribution from the anion species in the region 400–440 nm which is more pronounced for HBO-1, followed by HBO and HBO-2.

In order to correctly assign the different fluorescence signals to their corresponding tautomers, we measured the fluorescence lifetimes for the three probes in the four lipidic systems. Figure 3(b) displays the decay transients and the lifetime values are summarized in Table 1. It is important to mention that our lifetime assignments in lipids are based on the thoroughly studied dynamics of HBO in various solvents and incorporated inside DNA and protein^30^,^31^,^32^,^33^,^34^,^35^,^36^,^37^,^44^,^45^,^48^. We start with the fluorescence peak at 480 nm. This Stokes-shifted peak is due to the *syn*-keto tautomer that is formed after ESIPT. The lifetime of this tautomer falls in the range 0.5–1.1 ns (τ_2_ in the table) in all lipidic phases and for the three probes. This lifetime is far reduced from that measured in buffer for HBO and HBO-2, but within the same value as that of HBO-1 in buffer (comparing the transients for the probes in lipids with those of the probes in buffer only, shown in Figure 3(b)). This peak is then assigned to the closed *syn*-keto tautomer for all three probes. Our previous study on HBO in different solvents indicates that the lifetime of the closed *syn*-keto is sensitive to the solvent polarity^31^,^32^. It is clear that when the probes approach the head group region, the local environment is different from bulk water. This observation is an indication of the different environment experienced by the HBO moiety in the polar region of the head groups.

The current results complement earlier reports which show that water molecules tend to be more ordered and less flexible as they get closer to the head groups^49^. Using tryptophan and two of its derivatives, we have shown recently that the estimated polarity is less in the head group region compared to pure, bulk water^22^,^23^. The reduction in polarity in the head group region is due to the constrained water molecules which may explain the unique solvation characteristic of water that requires random distribution (disorder) of the water molecules in order to solvate the polar sites of the solute molecule, causing a maximum polarity effect. The less flexible water molecules in the head group region may interrupt the tendency of water molecules to strongly associate with each other through intermolecular hydrogen bonds that allow more than one molecule of water to form a solvent network^50^. Accordingly, the formation of the open *syn*-keto is not feasible when HBO is close to the head group region. The results also indicate that the majority of the HBO molecules form strong intramolecular hydrogen bonds that cannot be interrupted by the polar head groups as evident from the high intensity of the fluorescence peak of the closed *syn*-keto tautomer.

We used the two derivatives (HBO-1 and HBO-2) to probe different parts in the head group region. When a C_8_ alkyl chain is attached to the molecule, the HBO molecule is expected to be pulled closer to the polar head groups. This is due to the nature of the C_8_ chain being hydrophobic and tending to avoid the hydrophilic region. This tendency results in burying the chain inside the tail region of the lipid, which in turn brings the HBO molecule (attached to the chain) closer to the head groups. Similar spectral positions were obtained for the closed *syn*-keto tautomer when HBO, HBO-1, and HBO-2 were embedded in all the lipidic phases as shown in Figure 3(a), confirming the presence of a strong intramolecular hydrogen bond that is not affected by the polarity of the head groups. This observation indicates that the closed *syn*-keto tautomer is not sensitive to different locations within the head group region.

On the other hand, the fluorescence signal in the 370–380 nm region shows some variation in intensity for the different HBO molecules and in different lipids as shown in Figure 3(a). As indicated above, fluorescence in this spectral region is due to the solvated *syn*-enol and/or the *anti*-enol tautomers. The fluorescence lifetime values in Table 1 (τ_4_) indicate that this band is due to the solvated *syn*-enol only (the lifetime of the *anti*-enol tautomer is ~1.5 ns)^31^. We confirmed this assignment by measuring the fluorescence decay transients after eliminating any contribution from this band. The data in Table 1 shows that this lifetime component in all lipids (0.1–0.3 ns) cannot be detected when using a cut-off filter of 515 nm. Since this band is absent in buffer alone for all HBO derivatives (Figure 2(c)), it must derive from intermolecular interaction with the polar sites of the sugar units in lipids. Similar results were reported when HBO was used as a local reporter in a DNA duplex^48^,^51^ in which preferential stabilization of the solvated *syn*-enol tautomer was observed^48^. This was attributed to the formation of a hydrogen bond between the OH group of HBO and the O4′ atom of an adjacent nucleotide.

The results in Figure 3(a) imply that the two different orientations of the HBO moiety in HBO-1 and HBO-2 produce different concentrations of the solvated *syn*-enol species. This is explained by the fact that the formation of the solvated tautomer is expected to depend on how the OH group of HBO interacts with the polar sites of the head groups. In all the lipid phases, HBO-2 shows a smaller intensity in the 370–380 nm region compared to both HBO and HBO-1. This indicates that the OH group of the HBO molecule is oriented farther from the sugar units in HBO-2. The similarity between the spectra of HBO and HBO-1 in all lipidic phases implies that the HBO molecule in both cases is geometrically similar in the head group region. This is possible if the benzoxazole part of the molecule is closer to the head group sugar units. Given the geometry of the C_8_ chain in HBO-2, the OH group of HBO is expected to be oriented away from the sugar units. Accordingly, less solvation effect is expected as shown in Figure 3(a). This is reflected in the measured lifetime values shown in Table 1. The contribution from the solvated *syn*-enol is the smallest in HBO-2 compared with HBO and HBO-1 in all lipids. In one case, the lifetime component of the solvated *syn*-enol was not observed for HBO-2 imbedded in the hexagonal phase.

**Figure 4:**
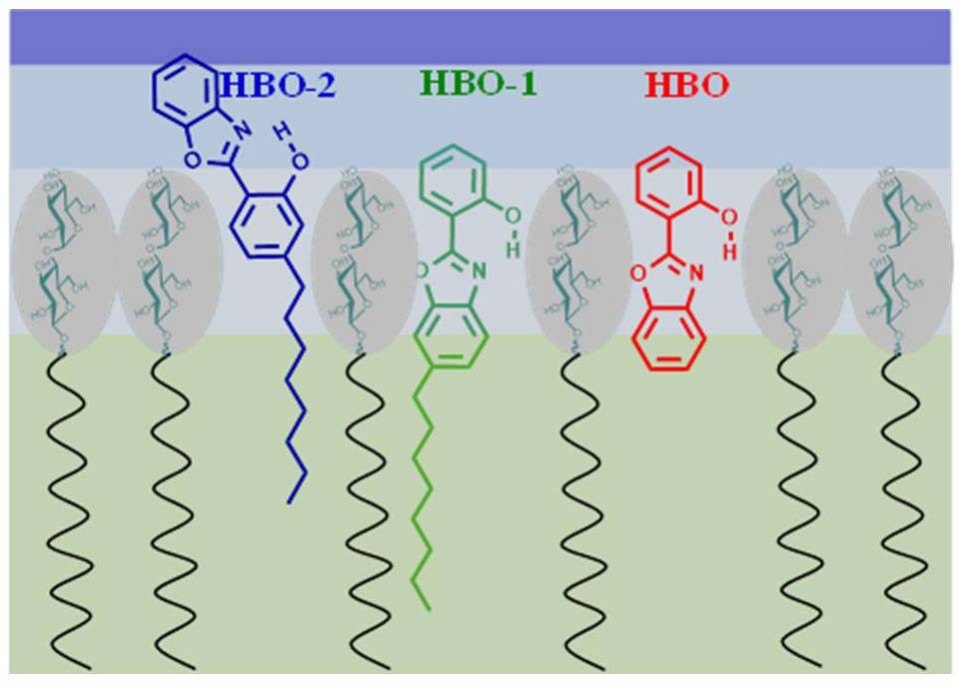
A proposed schematic diagram showing a layer of the *β*Mal-C_12_ lipid self-assembly with the expected locations of the probes. The blue region represents different layers of water structures. Dark blue: free water, ocean blue: restricted water, light blue: bounded water.

Comparing the fluorescence spectra in Figure 3(a) with those of HBO in different solvents (Figure 2(b)), the spectra in lipids resemble that of HBO dissolved in dioxane. This is in agreement with our recent results using tryptophan and its ester derivatives in which the local polarity was similar to that of dioxane when the tryptophan moiety was immersed deep in the head group region^22^,^23^. As mentioned above, the spectra shown in Figure 3(a) imply a similar location for HBO and HBO-1 within the head group region, while HBO-2 is slightly pushed away towards the restricted water region. Figure 4 shows the possible locations of the HBO probes. The association of the sugar group with the lipophilic moiety is made possible by the sugar amphoteric nature via the saccharide hydrophobic face^52^,^53^, thus stabilizing the mostly hydrophobic backbone of HBO. In a related study, Loura and Ramalho reported that when labelling 1,2-dipalmitoyl-*sn*-glycero-3-phosphocholine (DPPC) bilayers with 7-nitrobenz-2-oxa-1,3-diazol-4-yl (NBD) acyl-chain as a fluorescent probe, the NBD fluorophore adopts a transverse location closer to the water/lipid interface than to the center of the bilayer^54^. Since NBD moiety is closely related to HBO, we expect HBO to behave similarly and our results show that the probe moiety is located close to the sugar units. We should stress here on the fact that determining the exact location of the probes in the head group region is difficult with only experimental results. However, the relative orientation of the HBO molecule can be predicted from the difference in the spectral signals of the three probes as we discussed above.

Determining the location of the probes (at the surface or embedded in lipid) can also be estimated by performing a quenching experiment using for example acrylamide as a quencher of the probes' fluorescence (see our recent work on short peptides embedded in a bacterial membrane^55^). However, any collisional quenching experiment requires the quencher to rapidly diffuse and interact with the donor molecule during the lifetime of its excited state^56^. The rate of quenching is, therefore, inversely dependent on the viscosity of the medium through which diffusion occurs. Such experiments are not possible in the present work because all of the lipidic systems studied here exist in liquid crystalline phases with very high viscosity. A more insight into the structures of the probes/lipids systems may be obtained by performing molecular dynamics simulations. We expect the present results to be essential for such studies in order to correctly describe the systems.

### Detection of Anion Formation in the Head Group Region

A small contribution from the anion species of HBO is shown in Figure 3(a) in the spectral region 400–440 nm. As indicated before, no anion formation was observed for HBO and HBO-2 in buffer of pH 7.2, but it was detected for HBO-1 as a decay component in the fluorescence transient. Figure 3(a) shows the fluorescence spectrum of HBO in buffer in the upper segment of the graph for comparison. When we added maltose to HBO, we detected the anion signal as shown in the same graph (a blue tail in the spectral region 400–440 nm). This result points to a specific interaction that involves an intermolecular hydrogen bond between the OH group of HBO and a maltose oxygen atom that leads to a hydrogen ion abstraction. As shown in the figure, the anion contribution is much less for HBO-2 in all lipids compared to that of HBO and HBO-1. The trend is similar to that observed above for the solvated *syn*-enol tautomer. The latter may then act as an intermediate step that leads to ionization. The lifetime component for the anion species is shown in Table 1 for all lipids (τ_3_). This lifetime component is the longest among the three components in lipid and its value is overall longer than the lifetime of the anion species in buffer, indicating more stability in lipid. We confirmed this assignment by measuring the transient decay curves using a 515 nm cut-off filter. As shown in Table 1, the two lifetime components are for the anion (τ_3_) and the closed *syn*-keto (τ_2_), with a major contribution from the latter (compare with the same experiment when the observation window was ≥380 nm).

**Figure 5:**
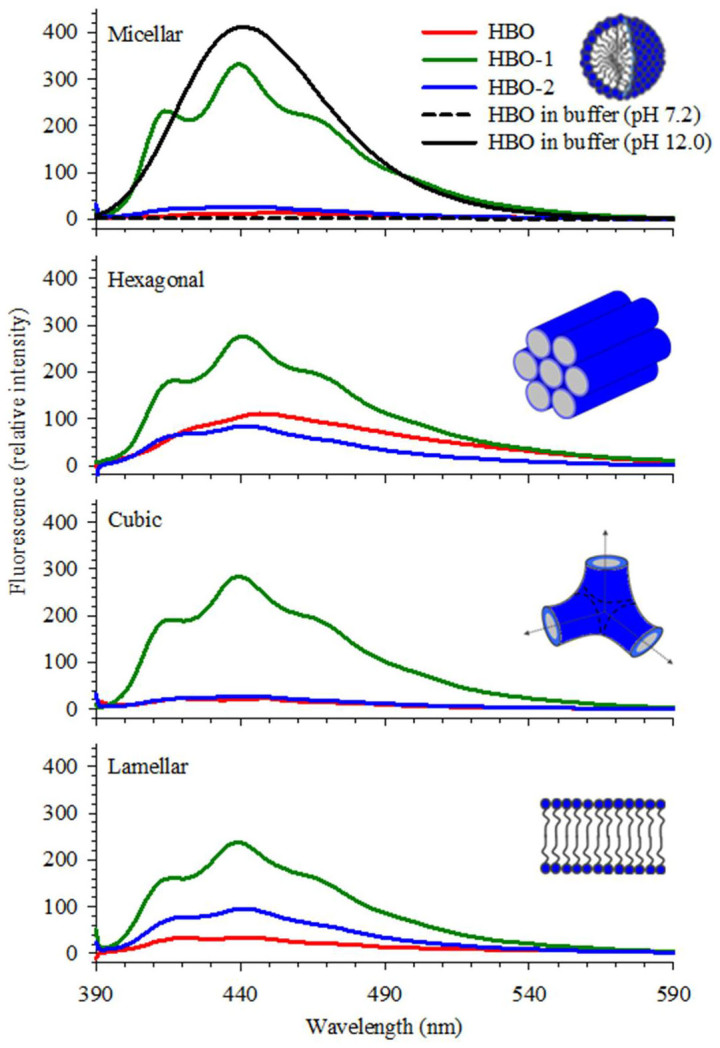
Fluorescence spectra of HBO and its derivatives in different phases of the *β*Mal-C_12_ lipid. λ_ex_ = 380 nm.

To clarify the anion contribution to the overall spectra in Figure 3(a), we measured the fluorescence signals after excitation at 380 nm where only the anion species absorbs (see Figure 2(a) for the absorption spectra of HBO). Figure 5 displays the fluorescence spectra, including those of HBO in buffers of pH 7.2 and 12.0 for comparison. It is clear that the anion spectrum of HBO-1 in all the lipidic phases is the most intense and structured which indicates a degree of restricted movement in the head group region. This restriction may stabilize the HBO moiety in a favored geometry that will enhance a hydrogen ion abstraction to yield the HBO anion.

The spectra in Figure 5 reveal some differences in the ionization strength in different lipidic phases. In general, HBO-1 shows the highest signal in all lipidic phases because of its high ability to form the anionic form as shown in Figure 2(c). For HBO and HBO-2, the anion signal in both the micellar and cubic phases is generally much less than that in the hexagonal and lamellar phases (Figure 5). This difference may correlate with the self-assembly nature of each two lipidic phases, being isotropic in the former two and anisotropic in the latter two. The anisotropic phase seems to enhance ionization.

It is worth mentioning here that liquid crystal formation was reported for a series of 2-phenylbenzoxazole derivatives containing an intramolecular hydrogen-bonded Schiff's base linkage^57^. The authors reported (with no assignment) similar structured fluorescence signals in the 400–450 nm region. We checked our probes using an optical polarizing microscope and we detected no signs of liquid crystal formation. The structured signals in Figure 3(a) are then a consequence of the effect of the surrounding lipid environment of different phases.

Membrane lipid component is one of the leading factors causing membrane heterogeneity at macroscopic and microscopic levels^58^. The results from our steady-state and time-resolved fluorescence provide further evidence to support this statement. Several tautomers of the probe systems were stable in different lipidic phases, due to the heterogeneous nature of the lipid self-assembly. Such heterogeneity is a key factor in accommodating molecules of different structures and sizes which what controls the biological function of the membrane as a whole. On the other hand, the ionization ability of the sugar unit, derived from its amphoteric nature, may be an inherited characteristic of any lipid self-assembly^59^,^60^. This is expected to affect the delivery of materials through the lipid bilayer such as transporting drugs^61^. Since most drugs are either weak acids or weak bases, the degree of ionization of the drug will be influenced by the polar head group units of any lipid.

## Conclusion

In summary, using HBO and two of its novel derivatives (HBO-1 and HBO-2) as fluorescent probes, we studied the microscopic nature of the head group region of the *β*Mal-C_12_ lipid self-assembly. The results in different lipidic phases (micellar, hexagonal, cubic and lamellar) show the stability of the solvated *syn*-enol, the anionic, and the closed *syn*-keto tautomers of HBO in all phases. The multi-tautomeric configurations of the HBO molecule in lipids reflect the heterogeneity nature of the head group region of the lipidic phases. This heterogeneity is related to the sugar amphoterism which is inherited in the lipid self-assembly and reflected in many biological functions. The detected HBO anions in all phases points to the ionization ability of the lipidic self-assembly which is also important to understand drug-lipid interaction and drug absorption by the cell membrane. Utilizing the spectroscopic sensitivity of HBO in the excited state to its local environment proved to be useful in the current study and we anticipate the HBO systems used here will be useful in other studies.

## Methods

### Synthesis of HBO-1 and HBO-2

Details of the novel synthetic procedures to prepare the HBO derivatives which include schemes, structure characterization and spectra can be found in the Supporting Information. We include here the final steps to prepare the probes (HBO-1 and HBO-2) used in the current study together with their ^1^H-NMR, ^13^C-NMR and GC/MS data. The compound numbers used here correspond to the schemes given in the Supporting Information.

### Preparation of HBO-1

2-(6-Octylbenzo[d]oxazol-2-yl)phenol (9). To a 100 mL flask, containing a solution of 802 mg (5.81 mmol, 2.00 eq.) salicylic acid in 40 mL dichloromethane, 500 μL (5.81 mmol, 2,00 eq.) oxalyl chloride and one drop DMF were added. After stirring the solution for 1.5 hours at ambient temperature the gas formation stopped. A solution of 765 mg (2.91 mmol, 1.00 eq.) 2-amino-5-octylphenyl acetate (**8**) and 1.78 mL (12.8 mmol, 4.40 eq.) NEt_3_ in 30 mL dichloromethane was added dropwise and the resulting solution was stirred at ambient temperature for 20 hours. The reaction was stopped by adding 30 mL H_2_O. After extraction with dichloromethane (3 × 30 mL) the combined organic phases were dried over Na_2_SO_4_ and the solvent was removed *in vacuo*. The residue was purified by silica gel chromatography (petroleum ether/diethyl ether, 5/1 → 2/1) to obtain 77.0 mg (0.201 mmol, 7%) of the desired amide 2-(2-hydroxybenzamido)-5-octylphenyl acetate, which was directly subjected to deprotection and condensation. A 10 mL flask, containing a solution of 77.0 mg (0.201 mmol, 1.00 eq.) 2-(2-hydroxybenzamido)-5-octylphenyl acetate and 19.1 mg (0.100 mmol, 0.50 eq.) *p*-toluenesulfonic acid monohydrate in 5 mL toluene, was heated to reflux for 20 hours. The reaction was allowed to cool to ambient temperature and the solvent was removed *in vacuo*. The residue was purified by silica gel chromatography (petroleum ether/diethyl ether, 4/1 → 2/1) to obtain 6.80 mg (0.021 mmol, 10%) of the title compound as a white solid.^**1**^**H-NMR** (400 MHz, CDCl_3_): *δ* = 8.01 (dd, *J* = 7.9, 1.5 Hz, 1H, *H*-13), 7.61 (d, *J* = 8.1 Hz, 1H, *H*-4), 7.46–7.40 (m, 2H, *H*-7, *H*-11), 7.21 (d, *J* = 8.1 Hz, 1H, *H*-5), 7.12 (d, *J* = 8.3 Hz, 1H, *H*-10), 7.01 (t, *J* = 7.6 Hz, 1H, *H*-12), 2.79–2.73 (m, 2H, *H*-14), 1.73–1.64 (m, 2H, *H*-15), 1.34–1.26 (m, 10H, *H*-16, *H*-17, *H*-18, *H*-19, *H*-20), 0.88 (t, *J* = 6.8 Hz, 3H, *H*-21). **^13^C-NMR** (126 MHz, CDCl_3_): *δ* = 162.4, 158.5, 149.4, 141.2, 138.0, 133.3, 126.9, 125.6, 119.5, 118.6, 117.3, 110.8, 110.1, 36.21, 31.86, 31.80, 29.45, 29.24, 29.21, 22.65, 14.09. **GC/MS (EI):** DB-50_L, t_r_ = 13.72 min; m/z = 323.30 [M].^+^. **C_21_H_25_NO_2_** (323.43).

### Preparation of HBO-2

2-(Benzo[d]oxazol-2-yl)-5-octylphenol (17).To an oven-dried 25 mL flask, containing a suspension of 184 mg (0.703 mmol, 3.00 eq.) PPh_3_ and 160 mg (0.703 mmol, 3.00 eq.) DDQ in 5 mL absolute toluene, a solution of 80.0 mg (0.234 mmol, 1.00 eq.) 2-hydroxy-*N*-(2-hydroxyphenyl)-4-octylbenzamide (**16**) in 5 mL absolute toluene was added. The resulting suspension was heated to reflux for 1.5 hours. The reaction was allowed to cool to ambient temperature and quenched by the addition of 15 mL H_2_O. After extraction with diethyl ether (3 × 15 mL) the combined organic phases were dried over Na_2_SO_4_ and the solvent was removed *in vacuo*. The residue was purified by silica gel chromatography (petroleum ether/diethyl ether, 50/1) to obtain 68.6 mg (0.212 mmol, 91%) of the title compound as a white solid.^**1**^**H-NMR** (300 MHz, CDCl_3_): *δ* = 7.92 (d, *J* = 8.1 Hz, 1H, *H*-13), 7.75–7.68 (m, 1H, *H*-7), 7.63–7.57 (m, 1H, *H*-4), 7.40–7.33 (m, 2H, *H*-5, *H*-6), 6.95 (d, *J* = 1.3 Hz, 1H, *H*-10), 6.84 (dd, *J* = 8.1, 1.5 Hz, 1H, *H*-12), 2.68 – 2.59 (m, 2H, *H*-14), 1.71–1.59 (m, 2H, *H*-15), 1.38–1.23 (m, 10H, *H*-16, *H*-17, *H*-18, *H*-19, *H*-20), 0.88 (t, *J* = 6.7 Hz, 3H, *H*-21). **^13^C-NMR** (75 MHz, CDCl_3_): *δ* = 163.1, 158.7, 149.8, 149.0, 140.0, 126.9, 125.1, 124.9, 120.2, 119.0, 117.0, 110.5, 108.1, 36.18, 31.86, 30.88, 29.44, 29.28, 29.22, 22.66, 14.10. **GC/MS (EI):** DB-50_L, t_r_ = 13.79 min; m/z = 323.30 [M].^+^. **C_21_H_25_NO_2_** (323.43).

### Materials and Preparation of Lipid-Probe Systems

*β*Mal-C_12_ (98%), HBO (98%) and D-Maltose (95%), anhydrous dioxane, DMSO, methanol and cyclohexane were all obtained from Sigma-Aldrich. All chemicals and solvents were used without further purification.

The concentration of HBO and its derivatives in the *β*Mal-C_12_/water system for both steady-state and time-resolved fluorescence experiments was adjusted to 0.1 mM. The value was based on an estimated density of ~1.0 g mL-1 for the mixture. Three sets of lipid/water systems were prepared. Each set includes the lipid mixed with the different probes, in addition to one sample with no probes, which was used as a standard in the measurements. The first set was a normal micellar phase with 40% (w/w) aqueous formulation at 23°C. The second set was a normal hexagonal phase with 65% (w/w) concentration at 23°C. The third set was a normal cubic phase with 80% (w/w) aqueous formulation at 50°C. This cubic phase is transformed into a lamellar phase when heated to 75°C. All phases were prepared according to the published binary phase diagram of *β*Mal-C_12_^40^ and were confirmed to be formed by illuminating the sample in the flame-sealed tube and examining them through a cross polarizing filter. The lamellar and hexagonal phases gave birefringent characteristic under the cross polarizing filter, which was not observed in the case of the micellar and cubic phases. Both micellar and cubic phases were optically isotropic under the cross-polarizing filter. All samples were prepared by mixing the lipids with HBO/HBO-1/HBO-2 dissolved in methanol. The methanol was evaporated afterward, and the samples were dried in a high vacuum to remove the solvent traces. A 40, 65, 80 mg amount of the mixture and 60, 35, 20 mg of buffer pH 7.2 were placed in a 4 mm diameter quartz tube for 40%, 65% and 80% (w/w) aqueous formulation respectively. The hydrated sample was immediately flame-sealed and underwent repeated cycles of centrifugation and heating to ensure that a homogeneous mixture was formed. The aqueous buffer used was 25 mM sodium phosphate buffer, pH 7.2. For HBO and its derivatives in solution, a stock solution in methanol (5 mM) was prepared. The solution was then diluted with buffer to reach the desired concentration. The final methanol:H_2_O (volume/volume) mixture was 5:95 which was shown to have pure water characteristics^37^,^62^. For basic solutions of HBO and its derivatives, pH 12.0 was achieved by adding aliquots of aqueous 1 M NaOH (Pellets, >99%, Sigma-Aldrich).

### Instrumentation

Absorption spectra were obtained with an Agilent 8453 Diode Array UV–vis spectrophotometer. Fluorescence spectra were recorded on a Shimadzu RF-5301 PC spectrofluorophotometer. Lifetime measurements were performed using a TimeMaster spectrofluorometer obtained from Photon Technology International. Excitation was done at 340 nm using light-emitting diodes. The instrument response function (IRF) was measured from the scattered light and estimated to be approximately 1.5 ns (full width at half-maximum).The measured transients were fitted to multiexponential functions convoluted with the system response function. The fit was judged by the value of the reduced chi-squared (χ^2^) which was close to 1.0 in all the fits. The experimental time resolution (after deconvolution) was approximately 100 ps, using stroboscopic detection^63^. In all the experiments, samples were measured in a 1 cm path-length quartz cell. The temperature of the samples was controlled within ±0.1°C at 23.0°C, 50.0°C, or 75.0°C. All samples were equilibrated overnight in the water bath at the desired temperature to ensure that the required phase was obtained before the measurements.

## Author Contributions

O.K.A.-Z. and R.H. designed and wrote the paper. N.I.Z. prepared the samples and performed the experiments. M.F.K. helped with some experimental measurements. D.S.G. and J.C.T. synthesized HBO-1 and HBO-2 molecules.

## Supplementary Material

Supplementary Information Supplementary Information

## Figures and Tables

**Table 1:** Summary of the fluorescence lifetime measurements of HBO and its derivatives in solution and incorporated in different phases of the *β*Mal-C_12_ lipid

		Lifetime (τ)			
Probe	λ_detection_	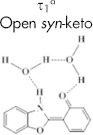	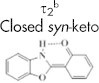	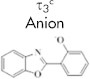	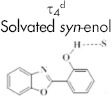
HBO in buffer pH 7.2	≥380 nm	5.5			
HBO in buffer pH 12.0	≥380 nm			2.6	
HBO-1 in buffer pH 7.2	≥380 nm		0.8 (0.51)	2.4 (0.49)	
HBO-1 in buffer pH 12.0	≥380 nm			2.8 (0.35)	0.4 (0.65)
HBO-2 in buffer pH 7.2	≥380 nm	6.1			
HBO-2 in buffer pH 12.0	≥380 nm	6.1 (0.74)		1.3 (0.26)	
Micellar phase					
HBO	≥380 nm		1.0 (0.35)	3.8 (0.24)	0.2 (0.41)
	≥515 nm		1.0 (0.60)	3.8 (0.40)	
HBO-1	≥380 nm		1.1 (0.35)	3.3 (0.22)	0.2 (0.43)
	≥515 nm		1.1 (0.76)	3.3 (0.24)	
HBO-2	≥380 nm		0.8 (0.35)	2.2 (0.45)	0.3 (0.20)
	≥515 nm		0.8 (0.78)	2.2 (0.22)	
Hexagonal phase					
HBO	≥380 nm		1.0 (0.31)	2.6 (0.35)	0.3 (0.34)
	≥515 nm		1.0 (0.63)	2.6 (0.37)	
HBO-1	≥380 nm		0.8 (0.39)	2.3 (0.47)	0.2 (0.14)
	≥515 nm		0.8 (0.70)	2.3 (0.30)	
HBO-2	≥380 nm		0.7 (0.37)	2.7 (0.63)	
	≥515 nm		0.7 (0.50)	2.7 (0.50)	
Cubic phase					
HBO	≥380 nm		0.6 (0.38)	4.7 (0.17)	0.1 (0.44)
	≥515 nm		0.6 (0.88)	4.7 (0.12)	
HBO-1	≥380 nm		0.8 (0.41)	5.1 (0.15)	0.2 (0.44)
	≥515 nm		0.8 (0.83)	5.1 (0.17)	
HBO-2	≥380 nm		0.6 (0.56)	3.8 (0.18)	0.3 (0.26)
	≥515 nm		0.6 (0.90)	3.8 (0.10)	
Lamellar phase					
HBO	≥380 nm		0.5 (0.47)	4.4 (0.16)	0.1 (0.37)
	≥515 nm		0.5 (0.89)	4.4 (0.11)	
HBO-1	≥380 nm		0.6 (0.49)	2.4 (0.18)	0.1 (0.33)
	≥515 nm		0.6 (0.90)	2.4 (0.10)	
HBO-2	≥380 nm		0.6 (0.75)	3.6 (0.15)	0.1 (0.10)
	≥515 nm		0.6 (0.85)	3.6 (0.15)	

Uncertainty in measurements is ^a^ ±0.2 ns; ^b^ ±0.4 ns; ^c^ ±0.8 ns; ^d^ ±0.1 ns. Relative contributions are listed in parentheses. Emission was detected using 380 and 515 nm cut-off filters. λ_ex_ = 340 nm.
