# Supplementary material for: Detecting local heterogeneity and ionization ability in the head group region of different lipidic phases using modified fluorescent probes
Source: Sci Rep. 2015 Mar 3;5:8699. doi: 10.1038/srep08699 (PMC4346976; doi:10.1038/srep08699)
Supplement: Supplementary Information [file srep08699-s1.pdf]

SUPPLEMENTARY INFORMATION

Detecting local heterogeneity and ionization ability in the head group region of different lipidic phases using modified fluorescent probes

Osama K. Abou-Zied[1,*] N. Idayu Zahid,[2] M. Faisal Khyasudeen,[2] David S. Giera,[3] Julian C. Thimm,[3] Rauzah Hashim,[2]

[1]Department of Chemistry, Faculty of Science, Sultan Qaboos University, P.O. Box 36, Postal Code 123, Muscat, Sultanate of Oman
[2]Department of Chemistry, Faculty of Science, University of Malaya, 50603 Kuala Lumpur, Malaysia
[3]Glycoteam GmbH, Martin-Luther-King-Platz 6, D-20146 Hamburg, Germany

*Corresponding Author: Osama K. Abou-Zied (E-mail: abouzied@squ.edu.om)

Table of Contents

Experimental section **S2**

Schemes **S2**

General Information **S3**

Procedures for the synthesis of HBO-1 (9) **S3 – S5**

Procedures for the synthesis of HBO-2 (17) **S6 – S8**

References **S9**

^{1}H-NMR, ^{13}C-NMR, ^{13}C-NMR (APT) and GC/MS spectra **S10 – S17**

Experimental Sections

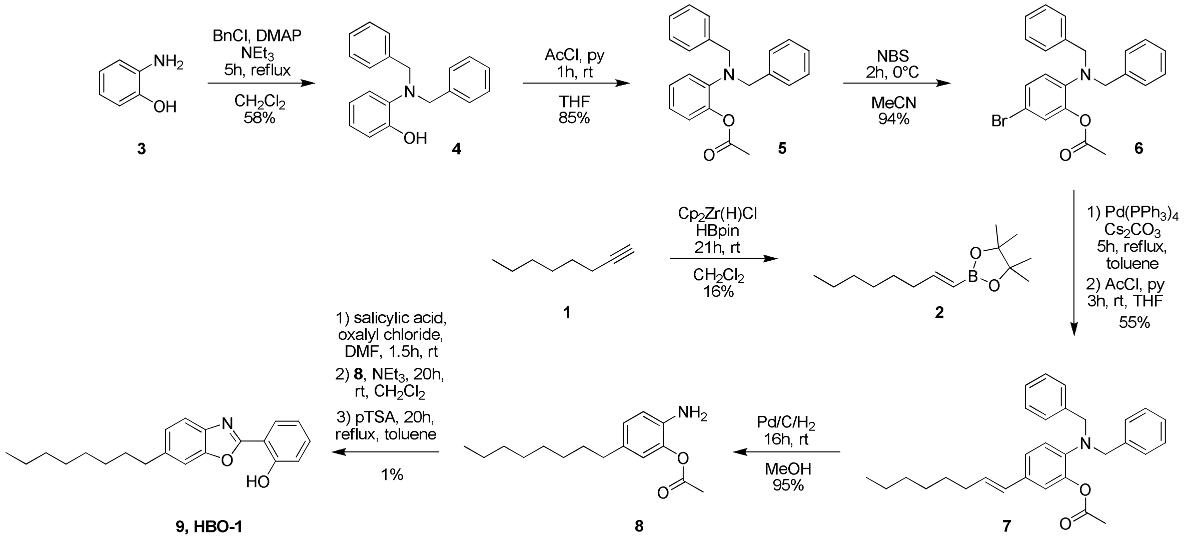

Scheme S1. Synthesis of HBO-1.

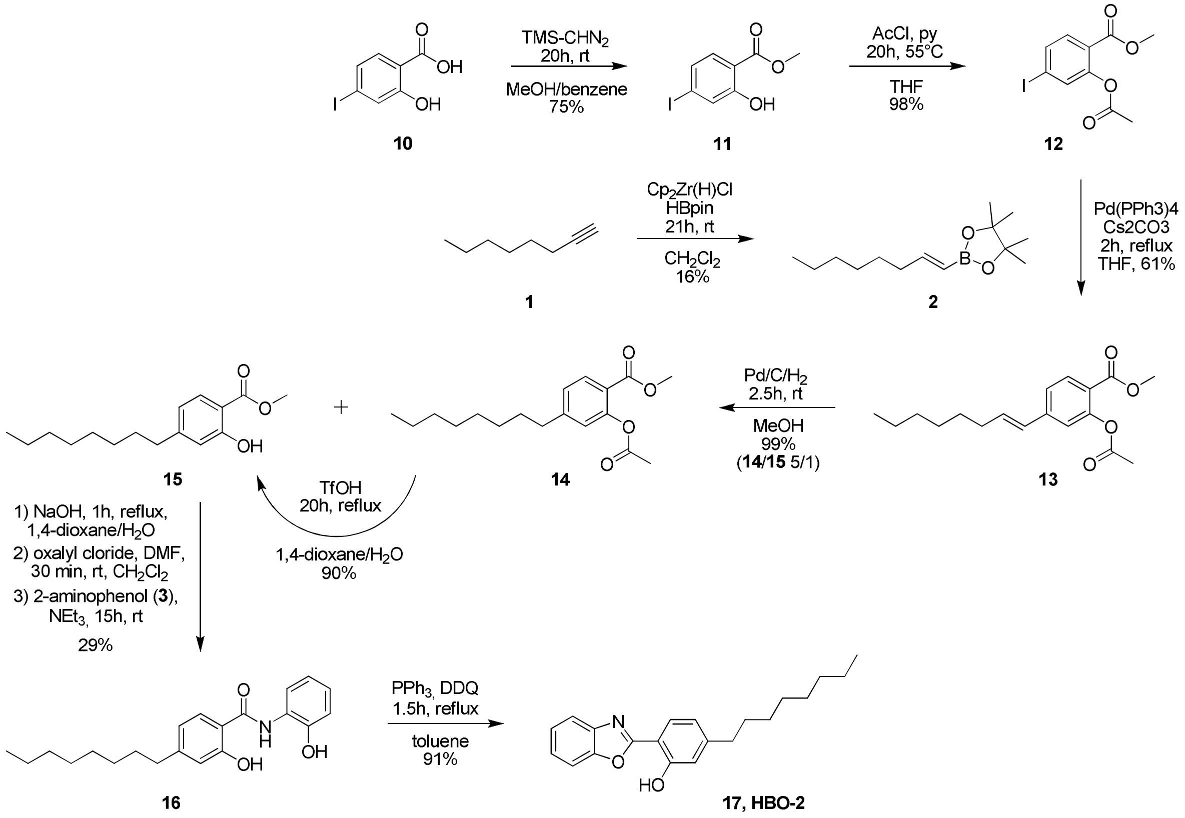

Scheme S2. Synthesis of HBO-2.

General Information

All reagents were used as purchased from commercial suppliers. Solvents were purified by conventional methods prior to use. Reactions were monitored by thin layer chromatography using Machery-Nagel pre-coated TLC-sheets ALUGRAM® Xtra SIL G/UV254 and visualized with potassium permanganate [potassium permanganate (2.4 g), K_2CO_3 (16 g), 5% NaOH (4.0 mL), H_2O (240 mL)], ceric ammonium molybdate [phosphomolybdic acid (25 g), $Ce(SO_4)_2 \cdot 2\ H_2O$ (10 g), H_2SO_4 conc. (60 mL), H_2O (940 mL)]. Chromatographic purification was performed as flash chromatography on Fluka silica gel 60 (particle size 0.040–0.063 mm). Yields refer to chromatographically purified and spectroscopically pure compounds. NMR spectra were recorded on a Bruker AV-400 (operating at 400 MHz for ^{1}H and 100 MHz for ^{13}C acquisitions), a Bruker AV-2400 (operating at 400 MHz for ^{1}H and 100 MHz for ^{13}C acquisitions), or a Bruker DRX-500 (operating at 500 MHz for ^{1}H and 125 MHz for ^{13}C acquisitions). Chemical shifts $\delta$are reported in ppm with the solvent resonance as the internal standard: chloroform-d1: 7.26 (^{1}H-NMR), 77.00 (^{13}C-NMR). Coupling constants *J* are given in Hertz (Hz). Multiplicities are classified as follows: s = singlet, d = doublet, t = triplet, q = quartet, sept = septet and combinations thereof, or m = multiplet or br = broad signal. Analytical gas chromatography was performed on a Varian CP-3800 with a flame ionization detector (FID) and a Varian autosampler (CP-8400) using a capillary column (WCOT Fused Silica 15 m x 0.25 mm, 0.25 µm CP-SIL 5CB) and nitrogen as carrier gas. High resolution mass spectra were obtained on a FinniganThermoQuest MAT 95XL. IR spectra were obtained on a Bruker ALPHA FT-IR Platinum ATR. Absorbance frequencies $\tilde{\nu}$ are reported in reciprocal centimeters (cm^{-1}).

Procedures

(*E*)-4,4,5,5-Tetramethyl-2-(oct-1-enyl)-1,3,2-dioxaborolane (2) [1]

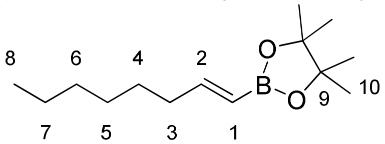

To a flame-dried 100 mL flask, containing a solution of 388 mg (95%, 1.43 mmol, 2 mol%) $Cp_2Zr(H)Cl$ and 11.1 mL (97%, 74.3 mmol, 1.05 eq.) pinacolborane in 40 mL absolute dichloromethane, 10.6 mL (99%, 70.8 mmol, 1.00 eq.) 1-octyne (**1**) was added slowly and the resulting solution was stirred for 21 hours at ambient temperature. The reaction was quenched by addition of 30 mL H_2O. The phases were separated and the aqueous phase was extracted with diethyl ether (3x 30 mL). The combined organic phases were washed with brine (1x 30 mL) and dried over Na_2SO_4 and the solvent was removed *in vacuo*. The residue was purified by silica gel chromatography (petroleum ether → petroleum ether/diethyl ether, 5/1) to afford 2.71 g (11.4 mmol, 16%) of the title compound as a colorless oil. **^{1}H-NMR** (300 MHz, $CDCl_3$): δ = 6.63 (dt, *J* = 17.9, 6.4 Hz, 1H, *H*-2), 5.42 (dt, *J* = 18.0, 1.5 Hz, 1H, *H*-1), 2.14 (td, *J* = 8.0, 1.5 Hz, 2H, *H*-3), 1.44 – 1.35 (m, 2H, *H*-4), 1.34 – 1.21 (m, 18H, *H*-5, *H*-6, *H*-7, *H*-10), 0.87 (t, *J* = 7.0 Hz, 3H, *H*-8). **^{13}C-NMR** (75 MHz, $CDCl_3$): δ = 154.9, 82.96, 35.84, 31.71, 28.91, 28.18, 24.77, 22.59, 14.09, *C*-1 not detected.
GC/MS (EI): DB-50_L, t_r = 8.75 min; m/z = 238.20 $[M]^{\cdot+}$. **$C_{14}H_{27}BO_2$** (238.17).

2-(Dibenzylamino)phenol (4)

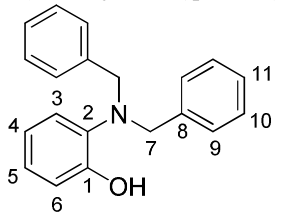

To an oven-dried 500 mL flask, containing a solution of 10.0 g (91.6 mmol, 1.00 eq.) 2-aminophenol (**3**), 1.12 g (9.16 mmol, 0.10 eq.) DMAP and 76.4 mL (550 mmol, 6.00 eq.) NEt_3 in 300 mL absolute dichloromethane, 52.7 mL (458 mmol, 5.00 eq.) benzyl chloride was added slowly at ambient temperature. The resulting solution was heated to reflux for 5 hours. The reaction was allowed to cool to ambient temperature and quenched by addition of 150 mL H_2O. The phases were separated and the aqueous phase was extracted with dichloromethane (3x 100 mL). The combined organic phases were dried over Na_2SO_4 and the solvent was removed *in vacuo*. The residue was purified by silica gel chromatography (petroleum ether/diethyl ether, 10/1, silica deactivation by 1% NEt_3 in the eluent) to afford 15.4 g (53.1 mmol, 58%) of the title compound as a colorless liquid. **^{1}H-NMR** (400 MHz, $CDCl_3$): δ = 7.31 – 7.16 (m, 10H, *H*-9, *H*-10, *H*-11), 7.13 (dd, *J* = 8.1, 1.3 Hz, 1H, *H*-4), 7.05 – 7.00 (m, 1H, *H*-5), 6.83 (d, *J* = 7.7 Hz, 2H, *H*-3, *H*-6), 4.02 (s, 4H, *H*-7). **GC/MS (EI):** DB-50_L, t_r = 11.71 min; m/z = 289.20 $[M]^{\cdot+}$. **$C_{20}H_{19}NO$** (289.37).

2-(Dibenzylamino)phenyl acetate (5)

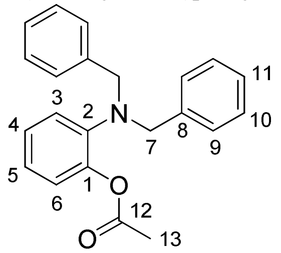

To an oven-dried 500 mL flask, containing a solution of 7.80 g (27.0 mmol, 1.00 eq.) 2-(dibenzylamino)phenol (**4**) in 200 mL absolute THF, 4.40 mL (53.9 mmol, 2.00 eq.) pyridine and 3.90 mL (98%, 53.9 mmol, 2.00 eq.) acetyl chloride were added consecutively. The resulting solution was stirred for 1 hour at ambient temperature, whereupon the reaction was quenched by addition of 100 mL sat. $NaHCO_3$-solution. After extraction with diethyl ether (3x 150 mL) the combined organic phases were washed with brine (1x 100 mL) and dried over Na_2SO_4 and the solvent was removed *in vacuo*. The residue was purified by silica gel chromatography (petroleum ether/diethyl ether, 6/1 → 5/1) to afford 7.58 g (22.9 mmol, 85%) of the title compound as a white solid. **^{1}H-NMR** (400 MHz, DMSO): δ = 7.32 – 7.24 (m, 8H, *H*-9, *H*-10), 7.24 – 7.18 (m, 2H, *H*-11), 7.08 – 6.98 (m, 2H, *H*-5, *H*-6), 6.97 – 6.90 (m, 2H, *H*-3, *H*-4), 4.21 (s, 4H, *H*-7), 3.31 (s, 3H, *H*-13). **^{13}C-NMR** (100 MHz, DMSO): δ = 168.9, 143.8, 142.6, 138.2, 128.2, 127.6, 126.8, 126.0, 123.7, 122.2, 121.7, 55.59, 20.66. **GC/MS (EI):** DB-50_L, t_r = 12.16 min; m/z = 331.20 $[M]^{\cdot+}$. **$C_{22}H_{21}NO_2$** (331.41).

5-Bromo-2-(dibenzylamino)phenyl acetate (6) [2]

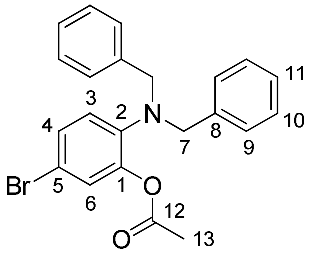

An oven-dried 500 mL flask, containing a solution of 7.58 g (22.9 mmol, 1.00 eq.) 2-(dibenzylamino)phenyl acetate (**5**) in 200 mL absolute acetonitril, was cooled to 0°C. A solution of 4.88 g (27.5 mmol, 1.20 eq.) NBS in 200 mL absolute acetonitril was added slowly. The resulting solution was stirred for 2 hours at 0°C, whereupon the reaction was quenched by addition of 100 mL H_2O. After extraction with petroleum ether (3x 150 mL) the combined organic phases were washed with brine (1x 100 mL) and dried over Na_2SO_4 and the solvent was removed *in vacuo*. The residue was purified by silica gel chromatography (petroleum ether/diethyl ether, 5/1) to afford 8.80 g (21.4 mmol, 94%) of the title compound as a white solid. **^{1}H-NMR** (300 MHz, DMSO): δ = 7.39 – 7.11 (m, 12H, *H*-4, *H*-6, *H*-9, *H*-10, *H*-11), 6.85 (d, *J* = 8.7 Hz, 1H, *H*-3), 4.23 (s, 4H, *H*-7), 2.10 (s, 3H, *H*-13). **^{13}C-NMR** (75 MHz, DMSO): δ = 168.8, 144.1, 142.2, 137.9, 128.8, 128.3, 127.5, 127.0, 126.7, 123.2, 112.5, 55.51, 20.62. **GC/MS (EI):** DB-50_L, t_r = 13.38 min; m/z = 409.20; 411.20 $[M]^{\cdot+}$. **$C_{22}H_{20}BrNO_2$** (410.30).

(*E*)-2-(Dibenzylamino)-5-(oct-1-enyl)phenyl acetate (7) [3-5]

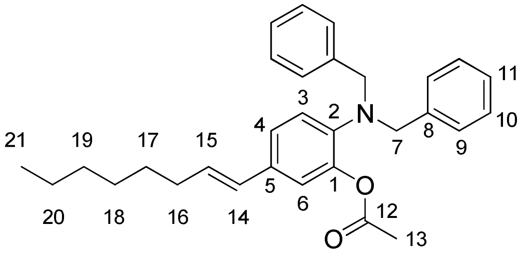

A flame-dried 100 mL flask, containing a solution of 2.30 g (5.61 mmol, 1.00 eq.) 5-bromo-2-(dibenzylamino)phenyl acetate (**6**), 1.34 g (5.61 mmol, 1.0 eq.) (*E*)-4,4,5,5-tetramethyl-2-(oct-1-enyl)-1,3,2-dioxaborolane (**2**), 324 mg (0.280 mmol, 5 mol%) $Pd(PPh_3)_4$ and 5.48 g (16.8 mmol, 3.00 eq.) cesium carbonate in 60 mL absolute degassed toluene, was heated to reflux for 5 hours. TLC-analysis indicated a partial loss of the acetyl protecting group of the coupling product. The reaction was allowed to cool to ambient temperature und quenched by addition of 40 mL H_2O. After extraction with diethyl ether (3x 40 mL) the combined organic phases were dried over Na_2SO_4 and the solvent was removed *in vacuo*. The residue was purified by silica gel filtration (petroleum ether/diethyl ether, 20/1 → 10/1) to afford 1,77 g of a crude product which was directly subjected to re-acetylation. To an oven-dried 100 mL flask, containing a solution of the crude product in 55 mL absolute THF, 714 µL (8.84 mmol) pyridine and 641 µL (98%, 8.84 mmol) acetyl chloride were added consecutively. The resulting solution was stirred for 3 hours at ambient temperature, whereupon the reaction was quenched by addition of 40 mL sat. $NaHCO_3$-solution. After extraction with diethyl ether (3x 40 mL) the combined

organic phases were washed with brine (1x 40 mL) and dried over Na_2SO_4 and the solvent was removed *in vacuo*. The residue was purified by silica gel chromatography (petroleum ether/diethyl ether, 25/1 → 15/1) to afford 1.36 g (3.09 mmol, 55%) of the title compound as a colorless oil. **^{1}H-NMR** (300 MHz, $CDCl_3$): δ = 7.33 – 7.21 (m, 10H, *H*-9, *H*-10, *H*-11), 7.06 – 6.97 (m, 2H, *H*-4, *H*-6), 6.79 (d, *J* = 8.1 Hz, 1H, *H*-3), 6.26 (d, *J* = 15.8 Hz, 1H, *H*-14), 6.08 (dt, *J* = 15.8, 6.8 Hz, 1H, *H*-15), 4.24 (s, 4H, *H*-7), 2.17 (td, *J* = 7.2, 6.8 Hz, 2H, *H*-16), 2.12 (s, 3H, *H*-13), 1.48 – 1.37 (m, 2H, *H*-17), 1.35 – 1.27 (m, 6H, *H*-18, *H*-19, *H*-20), 0.91 – 0.87 (m, 3H, *H*-21). **^{13}C-NMR** (75 MHz, $CDCl_3$): δ = 169.5, 143.7, 141.9, 138.1, 132.6, 130.3, 128.5, 128.3, 128.0, 126.9, 124.0, 121.6, 120.6, 55.64, 32.97, 31.74, 29.39, 28.87, 22.62, 20.93, 14.11. **GC/MS (EI):** DB-100_L, t_r = 12.07 min; m/z = 441.40 $[M]^{\cdot+}$. **$C_{30}H_{35}NO_2$** (441.60).

2-Amino-5-octylphenyl acetate (8)

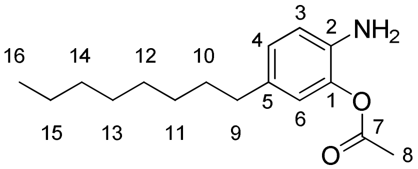

A flame-dried 250 mL flask, containing a solution of 1.36 g (3.09 mmol, 1.00 eq.) (*E*)-2-(dibenzylamino)-5-(oct-1-enyl)phenyl acetate (**7**) and 164 mg (1.54 mmol, 0.50 eq.) palladium on charcoal (10% Pd) in 50 mL absolute methanol was stirred rapidly. The flask was repeatedly evacuated and flushed with hydrogen and eventually set under a hydrogen atmosphere with a balloon. After 16 hours GC-MS analysis indicated complete conversion. The mixture was filtered through a pad of silica topped with celite. The solvent was removed *in vacuo* to afford 770 mg (2.92 mmol, 95%) of the title compound as a colorless oil. **^{1}H-NMR** (300 MHz, $CDCl_3$): δ = 8.85 (br s, 1H, N*H*), 7.87 (s, 1H, N*H*), 6.94 (d, *J* = 8.1 Hz, 1H, *H*-3), 6.82 (d, *J* = 1.6 Hz, 1H, *H*-6), 6.65 (dd, *J* = 8.1, 1.6 Hz, 1H, *H*-4), 2.59 – 2.42 (m, 2H, *H*-9), 2.22 (s, 3H, *H*-8), 1.63 – 1.47 (m, 2H, *H*-10), 1.26 (m, 10H, *H*-11, *H*-12, *H*-13, *H*-14, *H*-15), 0.87 (t, *J* = 6.7 Hz, 3H, *H*-16). **^{13}C-NMR** (75 MHz, $CDCl_3$): δ = 170.5, 148.3, 142.4, 123.2, 121.9, 120.5, 119.3, 35.26, 31.85, 31.19, 29.43, 29.22 (2C), 23.58, 22.64, 14.08. **MS (ESI):** m/z = 264.20 $[M+H]^+$, 286.18 $[M+Na]^+$, 302.14 $[M+K]^+$. **$C_{16}H_{25}NO_2$** (263.38).

HBO-1

2-(6-Octylbenzo[d]oxazol-2-yl)phenol (9) [6-10]

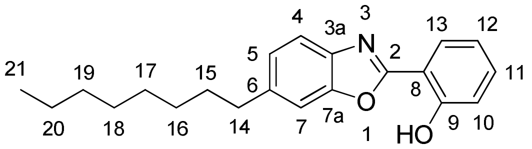

To a 100 mL flask, containing a solution of 802 mg (5.81 mmol, 2.00 eq.) salicylic acid in 40 mL dichloromethane, 500 µL (5.81 mmol, 2,00 eq.) oxalyl chloride and one drop DMF were added. After stirring the solution for 1.5 hours at ambient temperature the gas formation stopped. A solution of 765 mg (2.91 mmol, 1.00 eq.) 2-amino-5-octylphenyl acetate (**8**) and 1.78 mL (12.8 mmol, 4.40 eq.) NEt_3 in 30 mL dichloromethane was added dropwise and the resulting solution was stirred at ambient temperature for 20 hours. The reaction was stopped by addition of 30 mL H_2O. After extraction with dichloromethane (3x 30 mL) the combined organic phases were dried over Na_2SO_4 and the solvent was removed *in vacuo*. The residue was purified by silica gel chromatography (petroleum ether/diethyl ether, 5/1 → 2/1) to afford 77.0 mg (0.201 mmol, 7%) of the desired amide 2-(2-hydroxybenzamido)-5-octylphenyl acetate, which was directly subjected to deprotection and condensation. A 10 mL flask, containing a solution of 77.0 mg (0.201 mmol, 1.00 eq.) 2-(2-hydroxybenzamido)-5-octylphenyl acetate and 19.1 mg (0.100 mmol, 0.50 eq.) *p*-toluenesulfonic acid monohydrate in 5 mL toluene, was heated to reflux for 20 hours. The reaction was allowed to cool to ambient temperature and the solvent was removed *in vacuo*. The residue was purified by silica gel chromatography (petroleum ether/diethyl ether, 4/1 → 2/1) to afford 6.80 mg (0.021 mmol, 10%) of the title compound as a white solid. **^{1}H-NMR** (400 MHz, $CDCl_3$): δ = 8.01 (dd, *J* = 7.9, 1.5 Hz, 1H, *H*-13), 7.61 (d, *J* = 8.1 Hz, 1H, *H*-4), 7.46 – 7.40 (m, 2H, *H*-7, *H*-11), 7.21 (d, *J* = 8.1 Hz, 1H, *H*-5), 7.12 (d, *J* = 8.3 Hz, 1H, *H*-10), 7.01 (t, *J* = 7.6 Hz, 1H, *H*-12), 2.79 – 2.73 (m, 2H, *H*-14), 1.73 – 1.64 (m, 2H, *H*-15), 1.34 – 1.26 (m, 10H, *H*-16, *H*-17, *H*-18, *H*-19, *H*-20), 0.88 (t, *J* = 6.8 Hz, 3H, *H*-21). **^{13}C-NMR** (126 MHz, $CDCl_3$): δ = 162.4, 158.5, 149.4, 141.2, 138.0, 133.3, 126.9, 125.6, 119.5, 118.6, 117.3, 110.8, 110.1, 36.21, 31.86, 31.80, 29.45, 29.24, 29.21, 22.65, 14.09. **GC/MS (EI):** DB-50_L, t_r = 13.72 min; m/z = 323.30 $[M]^{\cdot+}$. **$C_{21}H_{25}NO_2$** (323.43).

Methyl 2-hydroxy-4-iodobenzoate (11) [11]

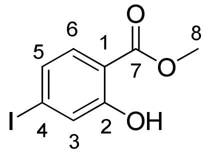

To an oven-dried 250 mL flask, containing a solution of 10.0 g (37.9 mmol, 1.00 eq.) 2-hydroxy-4-iodobenzoic acid (**10**) in 200 mL solvent mixture (methanol/benzene 5/3), 34.1 mL (2M in diethyl ether, 68.2 mmol, 1.80 eq.) (trimethylsilyl)diazomethane was added and the resulting solution was stirred for 20 hours at ambient temperature. The solvent was removed *in vacuo*. The residue was purified by silica gel chromatography (petroleum ether/ethyl acetate, 10/1 → 3/1) to afford 7.88 g (28.3 mmol, 75%) of the title compound as a white solid. **^{1}H-NMR** (400 MHz, $CDCl_3$): δ = 10.75 (s, 1H, O*H*), 7.50 (dd, *J* = 8.4, 0.7 Hz, 1H, *H*-5), 7.40 (s, 1H, *H*-3), 7.23 (d, *J* = 8.4 Hz, 1H, *H*-6), 3.94 (s, 3H, *H*-8). **^{13}C-NMR** (100 MHz, $CDCl_3$): δ = 170.3, 161.5, 130.6, 128.6, 127.0, 111.9, 102.7, 52.46. **$C_8H_7IO_3$** (278.04).

Methyl 2-acetoxy-4-iodobenzoate (12)

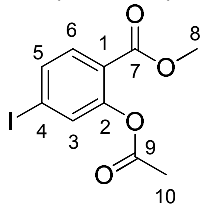

To an oven-dried 500 mL flask, containing a solution of 4.36 g (15.7 mmol, 1.00 eq.) methyl 2-hydroxy-4-iodobenzoate (**11**) in 250 mL absolute THF, 5.10 mL (62.7 mmol, 4.00 eq.) pyridine and 4.60 mL (98%, 62.7 mmol, 4.00 eq.) acetyl chloride were added consecutively. The resulting solution was heated to 55°C for 20 hours. The reaction was allowed to cool to ambient temperature and quenched by addition of 100 mL sat. $NaHCO_3$-solution. After extraction with diethyl ether (3x 150 mL) the combined organic phases were washed with brine (1x 100 mL) and dried over Na_2SO_4 and the solvent was removed *in vacuo*. The residue was purified by silica gel chromatography (petroleum ether/diethyl ether, 10/1 → 3/1) to afford 4.92 g (15.4 mmol, 98%) of the title compound as a white solid. **^{1}H-NMR** (400 MHz, $CDCl_3$): δ = 7.71 (d, *J* = 8.3 Hz, 1H, *H*-6), 7.66 (dd, *J* = 8.3, 1.6 Hz, 1H, *H*-5), 7.50 (d, *J* = 1.6 Hz, 1H, *H*-3), 3.86 (s, 3H, *H*-8), 2.34 (s, 3H, *H*-10). **^{13}C-NMR** (100 MHz, $CDCl_3$): δ = 169.3, 164.4, 150.7, 135.3, 133.1, 132.6, 122.8, 99.69, 52.31, 20.85. **GC/MS (EI):** DB-50_L, t_r = 9.90 min; m/z = 320.00 $[M]^{\cdot+}$. **$C_{10}H_9IO_4$** (320.08).

(*E*)-Methyl 2-acetoxy-4-(oct-1-enyl)benzoate (13) [3-5]

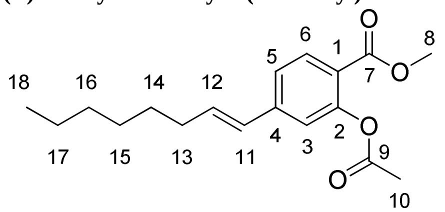

A flame-dried 25 mL flask, containing a solution of 130 mg (0.406 mmol, 1.00 eq.) methyl 2-acetoxy-4-iodobenzoate (**12**), 145 mg (0.609 mmol, 1.50 eq.) (*E*)-4,4,5,5-tetramethyl-2-(oct-1-enyl)-1,3,2-dioxaborolane (**2**), 46.9 mg (0.041 mmol, 10 mol%) $Pd(PPh_3)_4$ and 397 mg (1.22 mmol, 3.00 eq.) cesium carbonate in 12 mL absolute degassed THF was heated to reflux for 2 hours. The reaction was allowed to cool to ambient temperature und quenched by addition of 10 mL H_2O. After extraction with diethyl ether (3x 15 mL) the combined organic phases were dried over Na_2SO_4 and the solvent was removed *in vacuo*. The residue was purified by silica gel chromatography (petroleum ether/ethyl acetate, 10/1 → 8/1) to afford 74.9 mg (0.246 mmol, 61%) of the title compound as a colorless oil. **^{1}H-NMR** (400 MHz, $CDCl_3$): δ = 7.94 (d, *J* = 8.2 Hz, 1H, *H*-6), 7.24 (dd, *J* = 8.2, 1.7 Hz, 1H, *H*-5), 7.05 (d, *J* = 1.6 Hz, 1H, *H*-3), 6.37 – 6.33 (m, 2H, *H*-11, *H*-12), 3.85 (s, 3H, *H*-8), 2.35 (s, 3H, *H*-10), 2.22 (dt, *J* = 7.6, 6.3 Hz, 2H, *H*-13), 1.51 – 1.40 (m, 2H, *H*-14), 1.36 – 1.27 (m, 6H, *H*-15, *H*-16, *H*-17), 0.89 (t, *J* = 6.9 Hz, 3H, *H*-18). **^{13}C-NMR** (100 MHz, $CDCl_3$): δ = 169.8, 164.7, 151.1, 144.1, 135.5, 132.0, 128.1, 123.4, 120.7, 120.7, 52.02, 33.08, 31.68, 29.01, 28.87, 22.59, 21.01, 14.07. **GC/MS (EI):** DB-50_L, t_r = 11.56 min; m/z = 304.20 $[M]^{\cdot+}$. **$C_{18}H_{24}O_4$** (304.38).

Methyl 2-acetoxy-4-octylbenzoate (14) and Methyl 2-hydroxy-4-octylbenzoate (15)

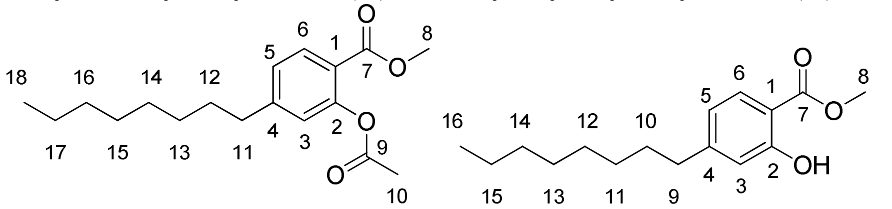

A flame-dried 100 mL flask, containing a solution of 347 mg (1.14 mmol, 1.00 eq.) (*E*)-methyl 2-acetoxy-4-(oct-1-enyl)benzoate (**13**) and 61.0 mg (0.570 mmol, 0.50 eq.) palladium on charcoal (10% Pd) in 20 mL absolute methanol was stirred rapidly. The flask was repeatedly evacuated and flushed with hydrogen and eventually set under a hydrogen atmosphere with a balloon. After 2.5 hours GC-MS analysis indicated complete conversion. The mixture was filtered through a pad of silica topped with celite. The solvent was removed *in vacuo* to afford 338 mg (1.13 mmol, 99%) as a 5/1 mixture of methyl 2-acetoxy-4-octylbenzoate (**14**) and methyl 2-hydroxy-4-octylbenzoate (**15**). Methyl 2-acetoxy-4-octylbenzoate (**14**)
^{1}H-NMR (300 MHz, $CDCl_3$): δ = 7.93 (d, *J* = 8.0 Hz, 1H, *H*-6), 7.11 (dd, *J* = 8.1, 1.7 Hz, 1H, *H*-5), 6.91 (d, *J* = 1.5 Hz, 1H, *H*-3), 3.85 (s, 3H, *H*-8), 2.68 – 2.59 (m, 2H, *H*-11), 2.35 (s, 3H, *H*-10), 1.67 – 1.55 (m, 4H, *H*-12, *H*-13), 1.34 – 1.23 (m, 8H, *H*-14, *H*-15, *H*-16, *H*-17), 0.88 (t, *J* = 6.7 Hz, 3H, *H*-18). **GC/MS (EI):** DB-50_L, t_r = 11.23 min; m/z = 264.20 $[M\text{-}Ac\text{+}H]^{\cdot+}$. **$C_{18}H_{26}O_4$** (306.40).
Methyl 2-hydroxy-4-octylbenzoate (**15**)
^{1}H-NMR (300 MHz, $CDCl_3$): δ = 10.71 (s, 1H, O*H*), 7.72 (d, *J* = 8.1 Hz, 1H, *H*-6), 6.80 (d, *J* = 1.4 Hz, 1H, *H*-3), 6.70 (dd, *J* = 8.2, 1.6 Hz, 1H, *H*-5), 3.93 (s, 3H, *H*-8), 2.63 – 2.53 (m, 2H, *H*-9), 1.66 – 1.57 (m, 2H, *H*-10), 1.34 – 1.22 (m, 10H, *H*-11, *H*-12, *H*-13, *H*-14, *H*-15), 0.87 (t, *J* = 6.8 Hz, 3H, *H*-16). **^{13}C-NMR** (75 MHz, $CDCl_3$): δ = 170.6, 161.6, 152.0, 129.7, 119.8, 117.0, 109.9, 52.09, 36.13, 31.85, 30.72, 29.40, 29.23, 29.20, 22.65, 14.09. **GC/MS (EI):** DB-50_L, t_r = 10.79 min; m/z = 264.20 $[M]^{\cdot+}$. **$C_{16}H_{24}O_3$** (264.36).

Methyl 2-hydroxy-4-octylbenzoate (15)

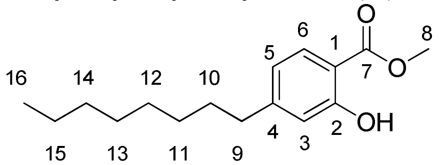

To a 25 mL flask, containing a solution of 328 mg (1.09 mmol, 1.00 eq.) of a 5/1 mixture of methyl 2-acetoxy-4-octylbenzoate (**14**) and methyl 2-hydroxy-4-octylbenzoate (**15**) in 12 mL solvent mixture (1,4-dioxan/H_2O 2/1), 144 µL (1.64 mmol, 1.50 eq.) trifluoromethanesulfonic acid was added. The resulting solution was heated to reflux for 20 hours. The reaction was allowed to cool to ambient temperature and 10 mL H_2O was added. After extraction with diethyl ether (3x 15 mL) the combined organic phases were dried over Na_2SO_4 and the solvent was removed *in vacuo*. The residue was purified by silica gel chromatography (petroleum ether/diethyl ether, 8/1 → 5/1) to afford 259 mg (0.980 mmol, 90%) of the title compound as a colorless oil. **^{1}H-NMR** (300 MHz, $CDCl_3$): δ = 10.71 (s, 1H, O*H*), 7.72 (d, *J* = 8.1 Hz, 1H, *H*-6), 6.80 (d, *J* = 1.4 Hz, 1H, *H*-3), 6.70 (dd, *J* = 8.2, 1.6 Hz, 1H, *H*-5), 3.93 (s, 3H, *H*-8), 2.63 – 2.53 (m, 2H, *H*-9), 1.66 – 1.57 (m, 2H, *H*-10), 1.34 – 1.22 (m, 10H, *H*-11, *H*-12, *H*-13, *H*-14, *H*-15), 0.87 (t, *J* = 6.8 Hz, 3H, *H*-16). **^{13}C-NMR** (75 MHz, $CDCl_3$): δ = 170.6, 161.6, 152.0, 129.7, 119.8, 117.0, 109.9, 52.09, 36.13, 31.85, 30.72, 29.40, 29.23, 29.20, 22.65, 14.09. **GC/MS (EI):** DB-50_L, t_r = 10.79 min; m/z = 264.20 $[M]^{\cdot+}$. **$C_{16}H_{24}O_3$** (264.36).

2-Hydroxy-*N*-(2-hydroxyphenyl)-4-octylbenzamide (16)

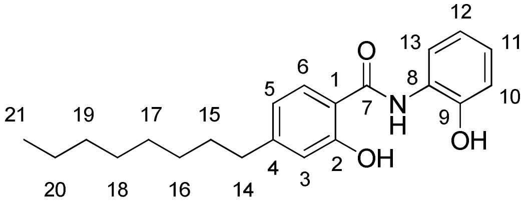

A 25 mL flask, containing a solution of 250 mg (0.946 mmol, 1.00 eq.) methyl 2-hydroxy-4-octylbenzoate (**15**) in 2 mL 1,4-dioxan and 6 mL 5N NaOH-solution, was heated to reflux for 1 hour. The reaction was allowed to cool to ambient temperature und quenched by addition of 15 mL 5N HCl-solution. After extraction with dichloromethane (3x 15 mL) the combined organic phases were washed with brine (1x 15 mL), dried over Na_2SO_4 and filtered. To the resulting solution 89 µL (1.04 mmol, 1,10 eq.) oxalyl chloride and 10 µL (0.129 mmol, 14 mol%) DMF were added. After stirring the solution for 30 min at ambient temperature the gas formation stopped. A solution of 207 mg (1.89 mmol, 2.00 eq.) 2-aminophenol (**3**) and 197 µL (1.42 mmol, 1.50 eq.) NEt_3 in 10 mL dichloromethane was added dropwise and the resulting solution was stirred at ambient temperature for 15 hours. The reaction was stopped by addition of 15 mL H_2O. After extraction with dichloromethane (3x 15 mL) the combined organic phases were dried over Na_2SO_4 and the solvent

was removed *in vacuo*. The residue was purified by silica gel chromatography (petroleum ether/diethyl ether, 5/1 → 2/1) to afford 92.6 mg (0.271 mmol, 29% over three steps) of the title compound as a colorless oil. **^{1}H-NMR** (300 MHz, $CDCl_3$): δ = 11.44 (s, 1H, O*H*), 8.32 (br s, 1H, N*H*), 7.59 (br s, 1H, O*H*), 7.47 (d, *J* = 8.2 Hz, 1H, *H*-6), 7.40 (d, *J* = 7.9 Hz, 1H, *H*-13), 7.16 (t, *J* = 7.7 Hz, 1H, *H*-11), 7.04 (d, *J* = 8.1 Hz, 1H, *H*-10), 6.96 (t, *J* = 7.6 Hz, 1H, *H*-12), 6.86 (s, 1H, *H*-3), 6.77 (dd, *J* = 8.2, 1.5 Hz, 1H, *H*-5), 2.65 – 2.56 (m, 2H, *H*-14), 1.67 – 1.58 (m, 2H, *H*-15), 1.32 – 1.25 (m, 10H, *H*-16, *H*-17, *H*-18, *H*-19, *H*-20), 0.88 (t, *J* = 6.7 Hz, 3H, *H*-21). **^{13}C-NMR** (75 MHz, $CDCl_3$): δ = 168.7, 161.5, 151.6, 148.2, 127.2, 125.9, 125.1, 122.6, 121.1, 120.0, 119.0, 118.5, 111.4, 35.96, 31.84, 30.70, 29.40, 29.21 (2C), 22.65, 14.09. **MS (ESI):** m/z = 342.21 $[M+H]^+$. **$C_{21}H_{27}NO_3$** (341.44).

HBO-2

2-(Benzo[d]oxazol-2-yl)-5-octylphenol (17) [6-10]

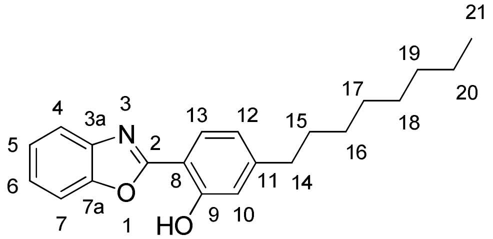

To an oven-dried 25 mL flask, containing a suspension of 184 mg (0.703 mmol, 3.00 eq.) PPh_3 and 160 mg (0.703 mmol, 3.00 eq.) DDQ in 5 mL absolute toluene, a solution of 80.0 mg (0.234 mmol, 1.00 eq.) 2-hydroxy-*N*-(2-hydroxyphenyl)-4-octylbenzamide (**16**) in 5 mL absolute toluene was added. The resulting suspension was heated to reflux for 1.5 hours. The reaction was allowed to cool to ambient temperature and quenched by addition of 15 mL H_2O. After extraction with diethyl ether (3x 15 mL) the combined organic phases were dried over Na_2SO_4 and the solvent was removed *in vacuo*. The residue was purified by silica gel chromatography (petroleum ether/diethyl ether, 50/1) to afford 68.6 mg (0.212 mmol, 91%) of the title compound as a white solid. **^{1}H-NMR** (300 MHz, $CDCl_3$): δ = 7.92 (d, *J* = 8.1 Hz, 1H, *H*-13), 7.75 – 7.68 (m, 1H, *H*-7), 7.63 – 7.57 (m, 1H, *H*-4), 7.40 – 7.33 (m, 2H, *H*-5, *H*-6), 6.95 (d, *J* = 1.3 Hz, 1H, *H*-10), 6.84 (dd, *J* = 8.1, 1.5 Hz, 1H, *H*-12), 2.68 – 2.59 (m, 2H, *H*-14), 1.71 – 1.59 (m, 2H, *H*-15), 1.38 – 1.23 (m, 10H, *H*-16, *H*-17, *H*-18, *H*-19, *H*-20), 0.88 (t, *J* = 6.7 Hz, 3H, *H*-21). **^{13}C-NMR** (75 MHz, $CDCl_3$): δ = 163.1, 158.7, 149.8, 149.0, 140.0, 126.9, 125.1, 124.9, 120.2, 119.0, 117.0, 110.5, 108.1, 36.18, 31.86, 30.88, 29.44, 29.28, 29.22, 22.66, 14.10. **GC/MS (EI):** DB-50_L, t_r = 13.79 min; m/z = 323.30 $[M]^{\cdot+}$. **$C_{21}H_{25}NO_2$** (323.43).

References

(1) Pereira, S.; Srebnik, M. *Organometallics* **1995**, *14*, 3127 – 3128.
(2) Zysman-Colman, E.; Arias, K.; Siegel, J. S. *Can. J. Chem.* **2009**, *87*, 440 – 447.
(3) (For reviews, see: (a) Miyaura, N.; Suzuki, A. *Chem. Rev.* **1995**, *95*, 2457 – 2483.
(4) Suzuki, A. *Angew. Chem., Int. Ed.* **2011**, *50*, 6722 – 6737.
(5) Heravi, M. M; Hashemi, E. *Tetrahedron* **2012**, *68*, 9145 – 9178).
(6) Quancai, X.; Zhengning, L.; Huiying, C. *Chin. J. Chem* **2011**, *29*, 925 – 932.
(7) Wang, B. B.; Maghami, N.; Goodlin, V. L.; Smith, P. J. *Bioorg. & Med. Chem. Lett.* **2004**, *14*, 3221 – 3226.
(8) Hunt, J. A.; Gonzales, S; Kallashi, F.; Hammond, M. L.; Pivnichny, J. V.; Tong, X.; Xu, S. S.; Anderson, M. S.; Chen, Y.; Eveland, S. S.; Guo, Q.; Hyland, S. A.; Milot, D. P.; Sparrow, C. P.; Wright, S. D.; Sinclaire, P. J. *Bioorg. & Med. Chem. Lett.* **2010**, *20*, 1019 – 1022.
(9) Huang, S.-T.; Hsei, I. -J.; Chen, C. *Bioorg. & Med. Chem. Lett.* **2006**, *14*, 6106 – 6119.
(10) Weidner-Wells, M. A.; Ohemeng, K. A.; Nguyen, V. N.; Fraga-Spano, S.; Macielag, M. J.; Werblood, H. M.; Foleno, B. D.; Webb, G. C.; Barrett, J. F.; Hlasta, D. J. *Bioorg. & Med. Chem. Lett.* **2001**, *11*, 1545 – 1548.
(11) Kühnel, E.; Laffan, D. D. P.; Lloyd-Jones, G. C.; Martínez del Campo, T.; Shepperson, I. R.; Slaughter, J. L. *Angew. Chem. Int. Ed.* **2007**, *46*, 7075 – 7078.

Spectra

HBO-1
2-(6-Octylbenzo[d]oxazol-2-yl)phenol (9)

^{1}H-NMR

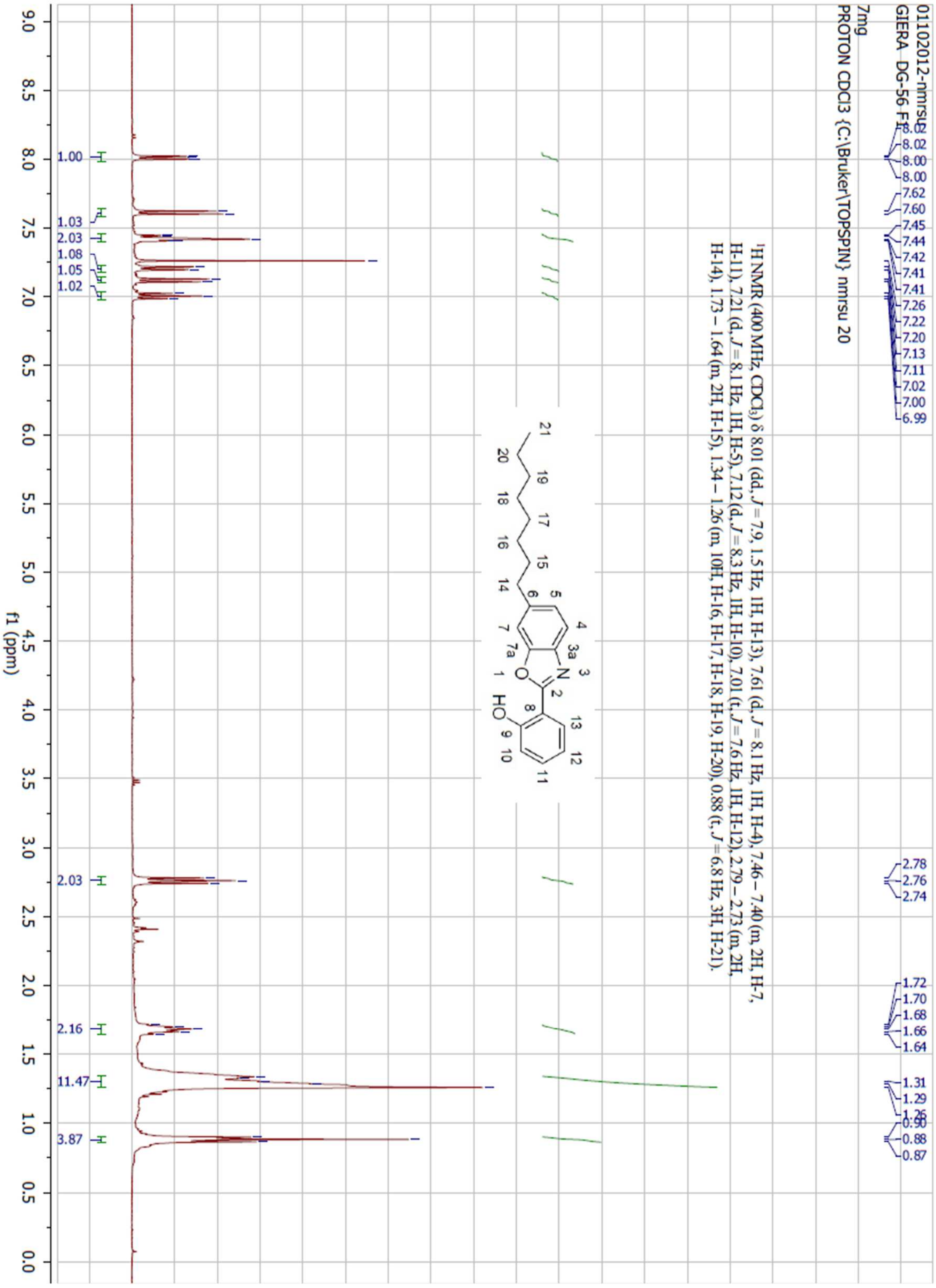

^{13}C-NMR

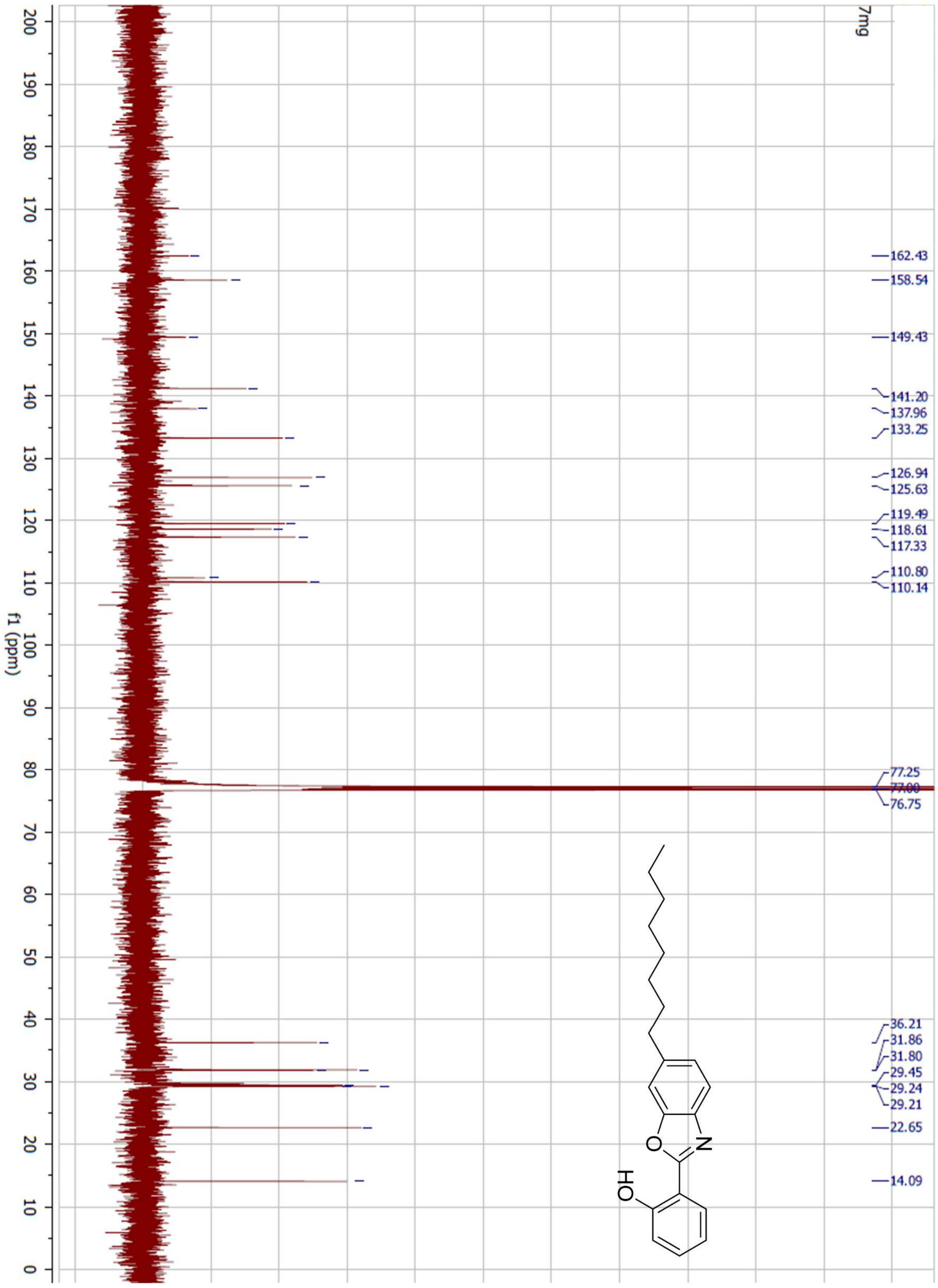
7mg
162.43
158.54
149.43
141.20
137.96
133.25
126.94
125.63
119.49
118.61
117.33
110.80
110.14
77.25
77.00
76.75
36.21
31.86
31.80
29.45
29.24
29.21
22.65
14.09
f1 (ppm)
HO
O
N

^{13}C-NMR (APT)

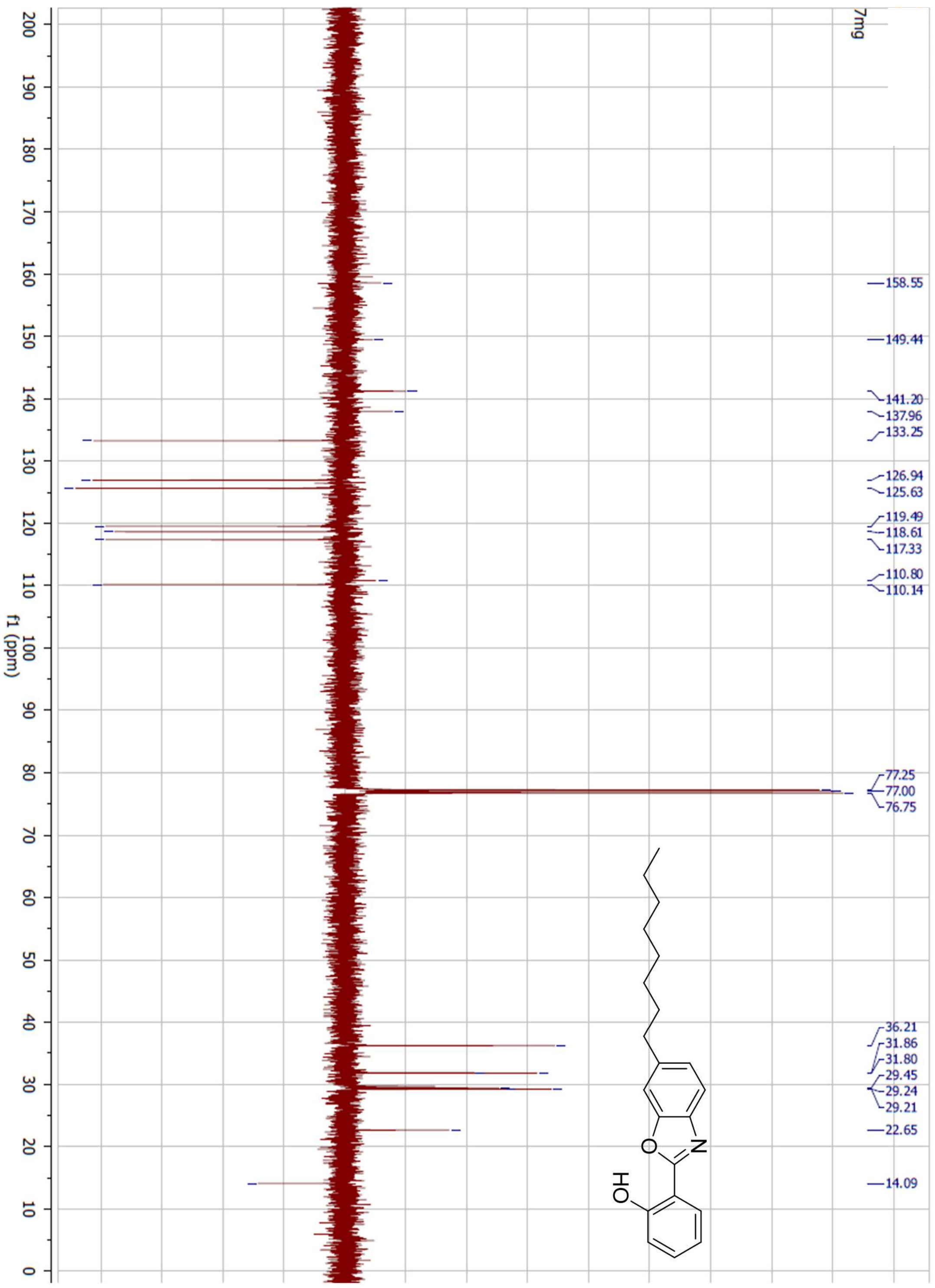

7mg
158.55
149.44
141.20
137.96
133.25
126.94
125.63
119.49
118.61
117.33
110.80
110.14
77.25
77.00
76.75
36.21
31.86
31.80
29.45
29.24
29.21
22.65
14.09
f1 (ppm)
HO
O
N

GC/MS

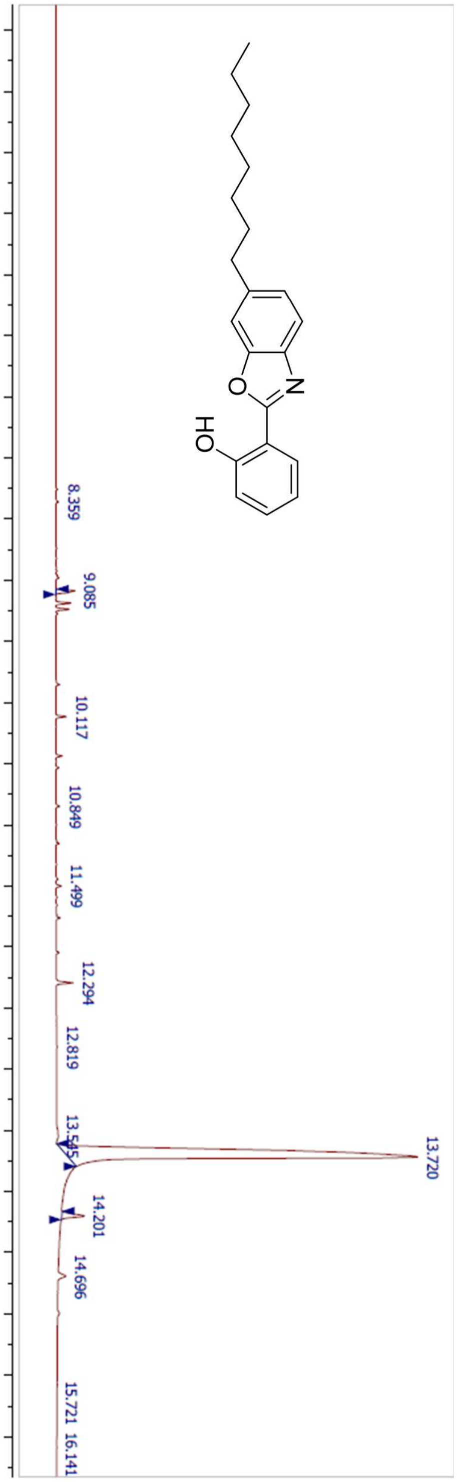

Retention time (min)
13.720
OH
N
O

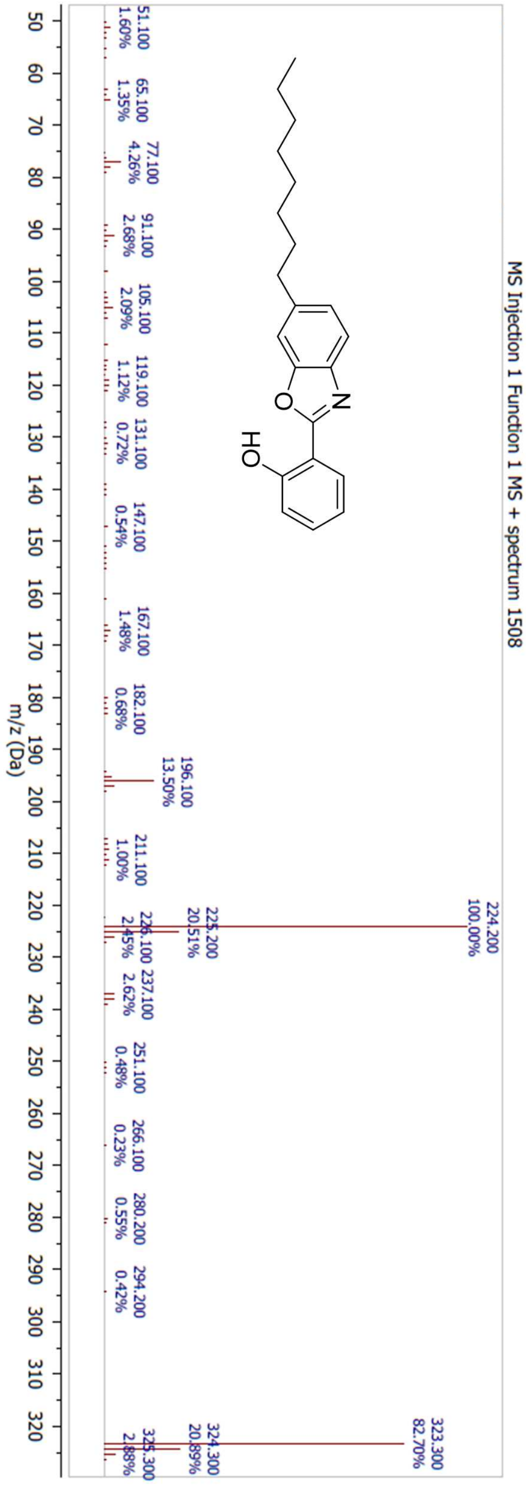

MS Injection 1 Function 1 MS + spectrum 1508
m/z (Da)
224.200
100.00%
323.300
82.70%
225.200
20.51%
324.300
20.89%
196.100
13.50%
OH
N
O

HBO-2
2-(Benzo[d]oxazol-2-yl)-5-octylphenol (17)

^{1}H-NMR

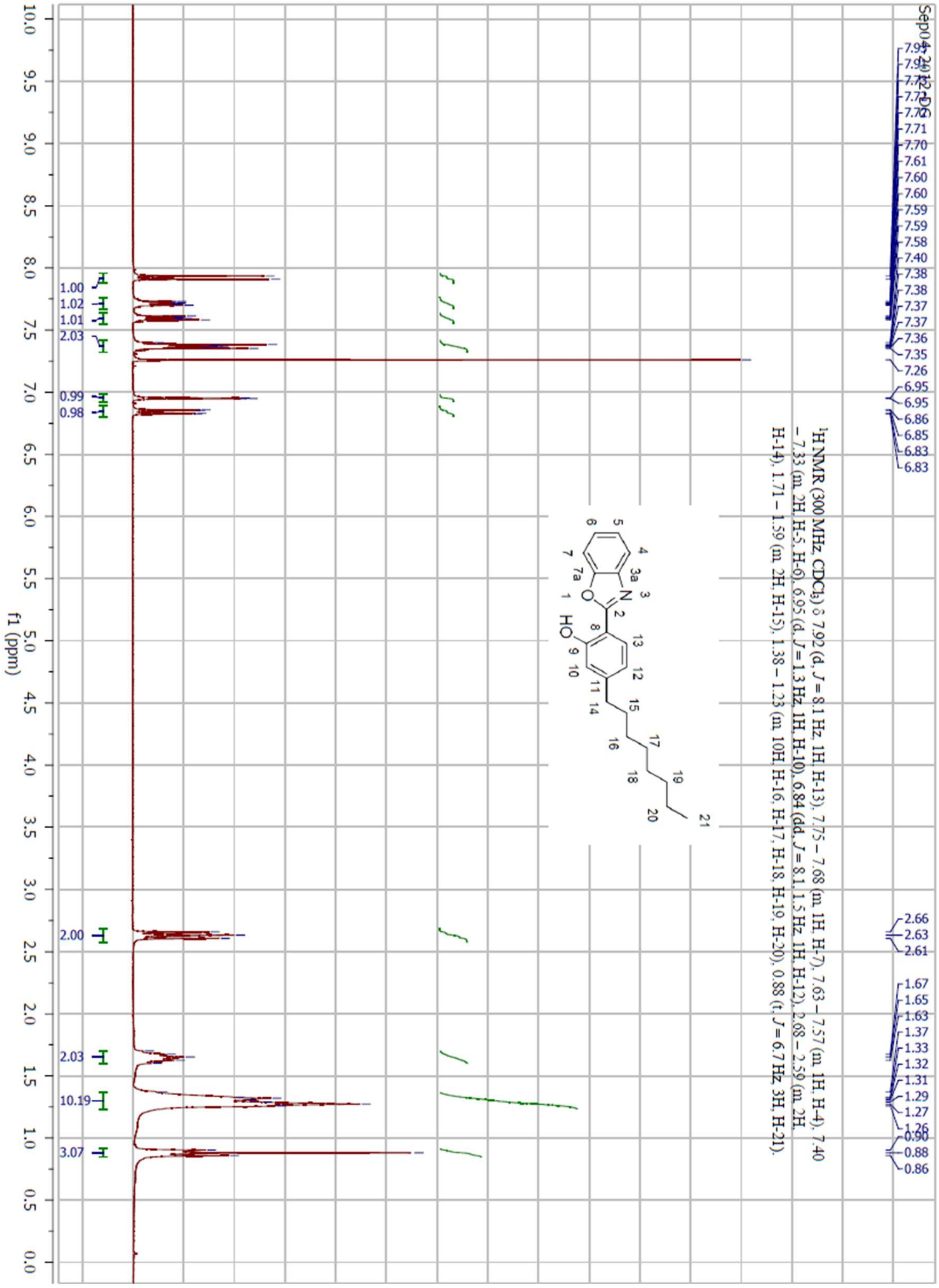

^{13}C-NMR

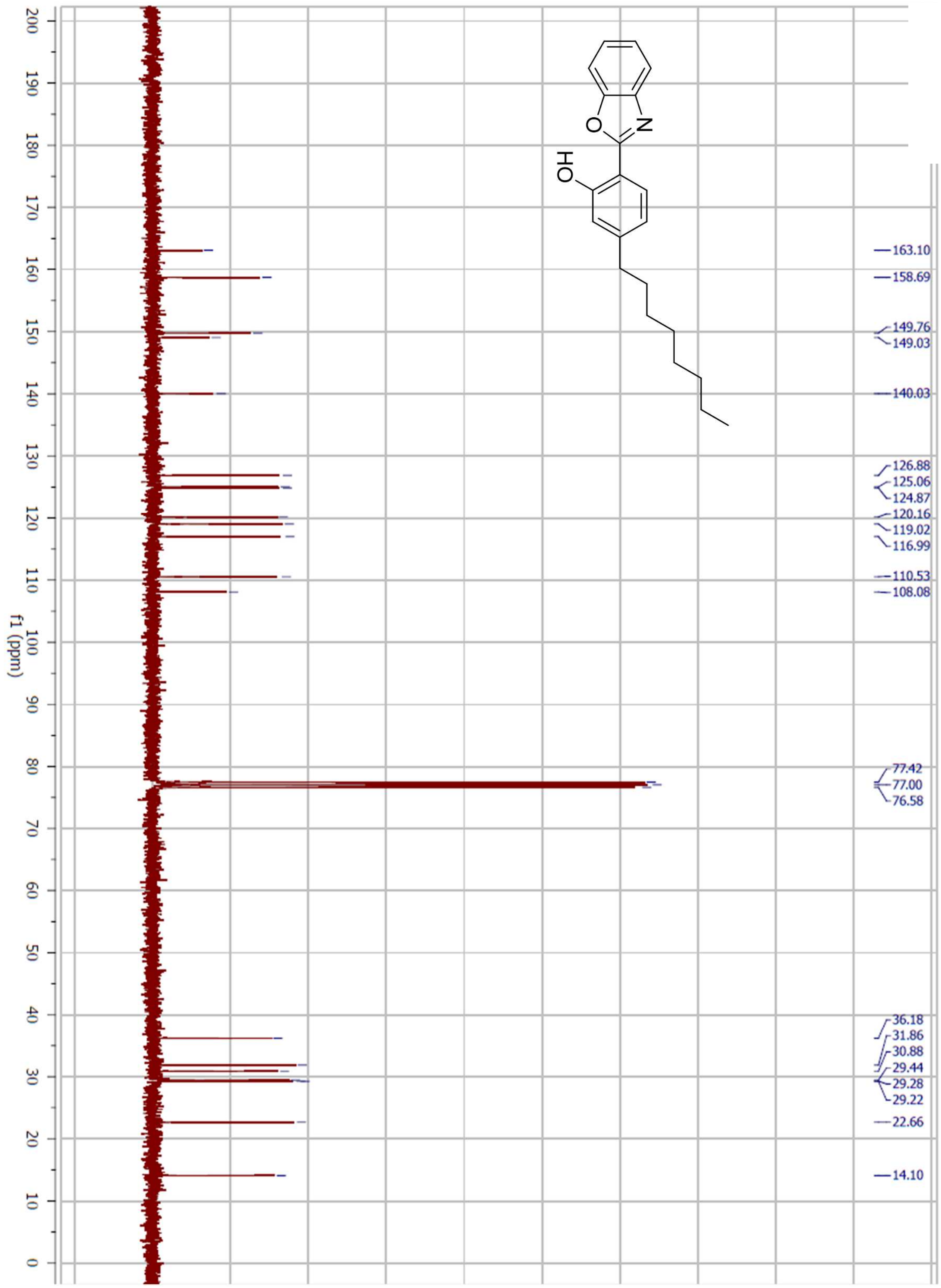

163.10
158.69
149.76
149.03
140.03
126.88
125.06
124.87
120.16
119.02
116.99
110.53
108.08
77.42
77.00
76.58
36.18
31.86
30.88
29.44
29.28
29.22
22.66
14.10
f1 (ppm)
OH
O
N

^{13}C-NMR (APT)

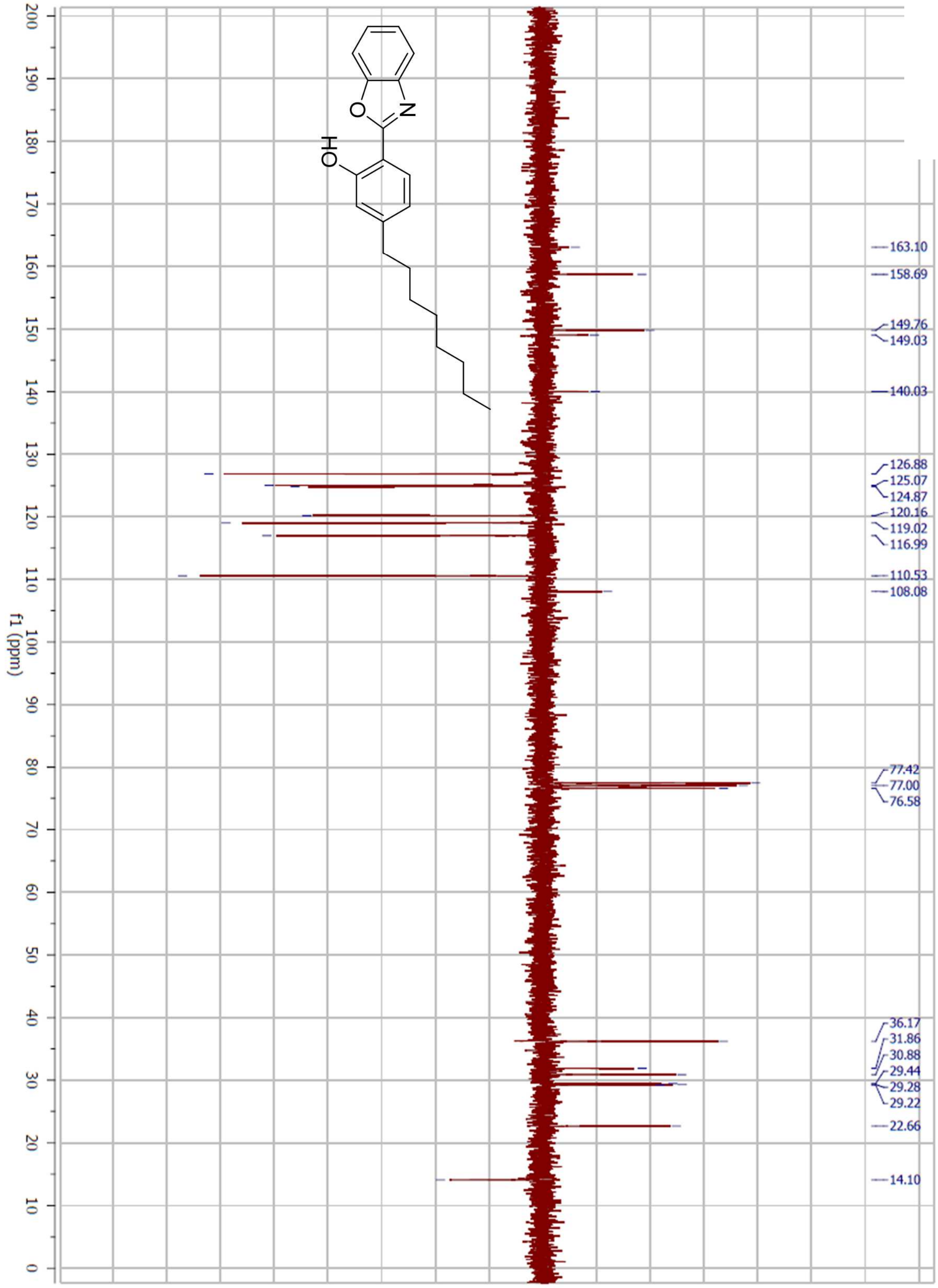
f1 (ppm)
163.10
158.69
149.76
149.03
140.03
126.88
125.07
124.87
120.16
119.02
116.99
110.53
108.08
77.42
77.00
76.58
36.17
31.86
30.88
29.44
29.28
29.22
22.66
14.10
OH
O
N

GC/MS

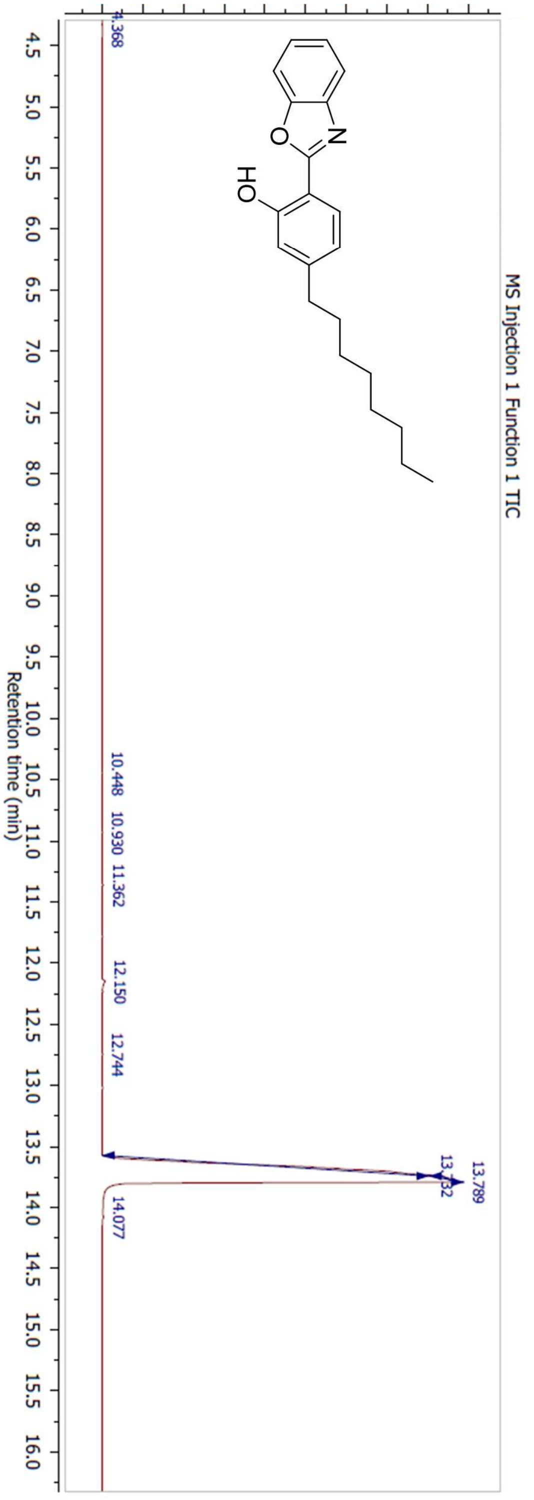

MS Injection 1 Function 1 TIC
HO
N
O
4.368
10.448
10.930
11.362
12.150
12.744
13.752
13.789
14.077
4.5
5.0
5.5
6.0
6.5
7.0
7.5
8.0
8.5
9.0
9.5
10.0
10.5
11.0
11.5
12.0
12.5
13.0
13.5
14.0
14.5
15.0
15.5
16.0
Retention time (min)

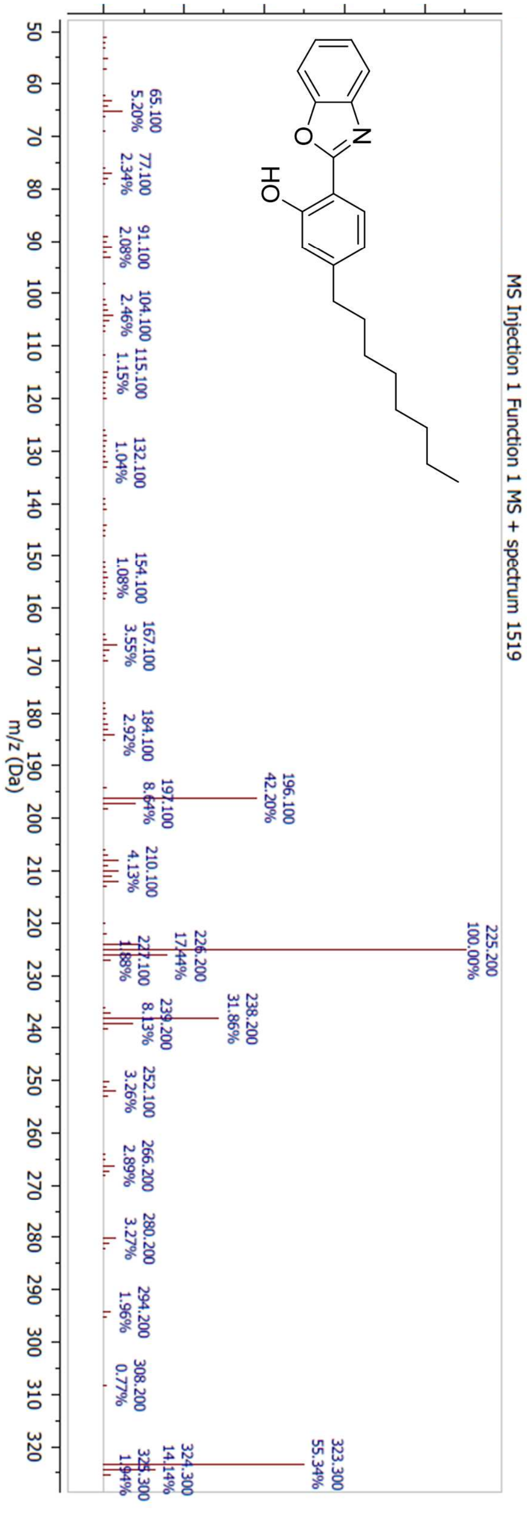

MS Injection 1 Function 1 MS + spectrum 1519
HO
N
O
65.100
5.20%
77.100
2.34%
91.100
2.08%
104.100
2.46%
115.100
1.15%
132.100
1.04%
154.100
1.08%
167.100
3.55%
184.100
2.92%
196.100
42.20%
197.100
8.64%
210.100
4.13%
225.200
100.00%
226.200
17.44%
227.100
1.88%
238.200
31.86%
239.200
8.13%
252.100
3.26%
266.200
2.89%
280.200
3.27%
294.200
1.96%
308.200
0.77%
323.300
55.34%
324.300
14.14%
325.300
1.94%
50
60
70
80
90
100
110
120
130
140
150
160
170
180
190
200
210
220
230
240
250
260
270
280
290
300
310
320
m/z (Da)
